# Quadrupedal training approaches in post-stroke rehabilitation: a scoping review of evidence, mechanisms, and clinical applications

**DOI:** 10.3389/fnsys.2026.1773330

**Published:** 2026-04-08

**Authors:** Jan A. Kuipers, Frederick R. Carrick, Monèm Jemni

**Affiliations:** 1 The Carrick Institute, Cape Canaveral, FL, United States; 2 Centre for Mental Health Research in Association with the University of Cambridge, Cambridge, United Kingdom; 3 College of Medicine, University of Central Florida, Orlando, FL, United States; 4 Burnett School of Biomedical Science, University of Central Florida, Orlando, FL, United States; 5 MGH Institute of Health Professions, Boston, MA, United States; 6 Faculty of Physical Education, Ningbo University, Ningbo, China

**Keywords:** stroke rehabilitation, quadrupedal training, trunk control, balance, central pattern generators, interlimb coupling, neuroplasticity

## Abstract

**Background:**

Persistent impairments in trunk control, balance, and mobility are frequently observed after stroke, even after standard task-specific rehabilitation. Quadrupedal-derived training (QT)—which involves four-point support, dynamic contralateral tasks, transitional kneeling, and crawling—has attracted clinical interest because it may activate bilateral and spinal sensorimotor networks. Nonetheless, the evidence supporting QT has not been thoroughly systematically mapped. Objective: To synthesize the extent, characteristics, mechanisms, and clinical applications of quadrupedal-derived training in adult post-stroke rehabilitation.

**Methods:**

A scoping review was conducted in accordance with the JBI Manual for Evidence Synthesis and the PRISMA-ScR guidelines. It involved searching five databases and additional sources from 2010 to 2025 to find studies on QT in stroke populations, along with mechanistic and translational evidence. The outcomes were pre-mapped to the International Classification of Functioning (ICF) domains. Data on intervention types, total dosage, supervision, progression criteria, safety, and feasibility were gathered. Stakeholder input from stroke survivors, clinicians, and researchers helped shape implementation considerations.

**Results:**

Eighteen studies met the inclusion criteria, including five randomized controlled trials and one case study involving stroke populations, as well as mechanistic and translational research. QT consistently improved trunk control and balance, with effects on functional mobility and certain gait parameters varying depending on the variant and dose. Kneeling-based QT showed greater balance benefits than treadmill-based training in subacute inpatient settings, while static and dynamic four-point variants were mainly used with chronic outpatient groups. No serious adverse events occurred, and adherence was high where recorded. Mechanistic evidence indicates a pathway connecting quadrupedal loading to activation of spinal and interlimb networks, bilateral proximal muscles, and functional improvements.

**Conclusion:**

Quadrupedal-based training is a biologically plausible, resource-efficient, and clinically practical method for improving trunk and balance issues after a stroke. More well-designed studies that include standardized progression, dose–response evaluations, and neurophysiological biomarkers are needed.

## 1 Introduction

Stroke remains a leading cause of long-term disability globally, and many survivors reach a plateau despite modern task-specific rehabilitation (Feigin et al., 2021; Langhorne et al., 2009; Stinear et al., 2020; Ward et al., 2019; Winters et al., 2018). These plateaus inspire strategies that engage underutilized neural resources and promote plasticity beyond typical bipedal practice. In this review, we progress from mechanisms to interventions, then outcomes, and finally feasibility and translation, concluding with gaps and future priorities. This approach aims to link biological plausibility with clinical application (Bernhardt et al., 2024; Langhorne et al., 2017).

Quadrupedal training (QT) is a form of rehabilitation that deliberately loads both upper and lower limbs simultaneously, typically in a four-point or transitional kneeling position. QT encompasses static four-point holds, dynamic contralateral activities such as bird-dog, rock- backs, and cat–camel, as well as kneeling or half-kneeling sequences. When tested, it also includes hands-and-knees locomotion. These specific forms are called QT variants.

Four-limb loading provides extensive somatosensory input and diagonal interlimb coupling at spinal levels, stimulating central pattern generators (CPGs) and long propriospinal pathways that link cervical and lumbar segments (Frigon, 2017; Dimitrijevic et al., 2024; Zehr and Duysens, 2004). Simultaneously, QT primarily activates bilateral proximal and trunk muscles, potentially promoting more symmetrical sensorimotor activation than bipedal tasks alone (Buxton et al., 2024; Fisher et al., 2024). Animal studies show that engaging forelimbs during quadrupedal step training reorganizes rostro-caudal propriospinal networks and enhances hindlimb coordination after hemisection (Shah et al., 2013). Clinical guidelines underscore the importance of proximal activation and aerobic/locomotor principles in post-stroke rehabilitation (MacKay-Lyons et al., 2023). Collectively, these mechanisms suggest a plausible pathway from QT to improvements in postural control, trunk stability, and walking efficiency.

Despite biological plausibility, specific clinical evidence for quadrupedal training (QT) in post- stroke populations remains limited but promising. An inpatient randomized trial found kneeling improved balance (BBS) more than treadmill walking and selectively increased paretic step length (Zhang et al., 2024). Outpatient trials of various QT variants have reported improvements in TIS, TUG, gait speed/cadence, and SS-QOL. A case report of chronic stroke also reports functional gains with a modified quadruped program (Pascal et al., 2022). Supporting evidence from related fields confirms feasibility and mechanistic plausibility, such as propriospinal engagement in SCI and synergistic recruitment in CP (Dietz, 2011; Prosser et al., 2010). Related pediatric neurorehabilitation literature also suggests that activity-oriented interventions can improve physical activity in early brain injury populations (Mitchell et al., 2012).

Lifespan studies of crawling suggest that coordination patterns remain accessible even after long periods of disuse (Cole et al., 2019). However, standardized definitions, dose parameters, and comparators are still lacking, and dose–response research remains limited (Lohse et al., 2014).

This scoping review charts the scope, variety, and characteristics of QT evidence in adult stroke rehabilitation, focusing on mechanisms, protocols/dose, outcomes (ICF domains), and implementation/feasibility, to establish an evidence-based foundation and highlight priorities for upcoming trials.

Using the JBI framework for scoping reviews (Munn et al., 2023) and following PRISMA-ScR guidelines (Tricco et al., 2018), this review explores the question: “What is the extent, range, and nature of evidence on quadrupedal training methods in post-stroke rehabilitation?”

To operationalize this, we explored four linked objectives:

1. What neurophysiological mechanisms, like CPG activation, propriospinal integration, and bilateral cortical recruitment, have been suggested to explain possible QT benefits?
2. Intervention characteristics: Which QT variants, progression strategies, and dosing parameters have been used across different stroke populations and clinical environments?
3. What clinical outcomes are reported across the ICF domains, body functions, activities, and participation?
4. Implementation and feasibility: What evidence exists regarding safety, adherence, resource needs, and how well it can be translated into practice?

## 2 Methods

### 2.1 Review design and reporting

We conducted a scoping review in accordance with the JBI Manual for Evidence Synthesis (Munn et al., 2023). We reported our use of the Preferred Reporting Items for Systematic Reviews and Meta-Analyses extension for Scoping Reviews (PRISMA-ScR) checklist.

### 2.2 Eligibility criteria

*Participants:* Adults (≥ 18 years) with ischemic or hemorrhagic stroke at any stage of recovery (acute: < 7 days; subacute: 7 days to 6 months; chronic: > 6 months post-stroke).

*Concept:* Interventions prescribing weight bearing through both upper and lower limbs in a four- point/quadruped position, with or without locomotion. Eligible variants included:

- Static quadruped positions (hands-and-knees holds, weight shifts, rock-backs)
- Dynamic quadruped tasks (contralateral arm/leg raises, bird-dog exercises, perturbation tasks)
- Quadrupedal locomotion (hands-and-knees crawling, bear-crawl, modified crawling patterns)
- Closely related transitions when quadruped was a planned, dose-bearing component
- Kneeling-based training protocols incorporating quadrupedal elements

For clarity, the review separated low quadruped positions (hands-and-knees or four-point support with hips flexed) from tall kneeling positions (hips extended, knees on the ground). Studies were considered eligible if they included tall-kneeling or half-kneeling sequences as part of transitional or weight-bearing quadruped variants; however, isolated tall-kneeling balance or postural exercises without upper-limb weight-bearing were excluded.

*Comparators:* Any (usual care, other therapies, sham, none).

*Outcomes:* Any outcome measure mapped to the ICF framework or implementation outcomes (adverse events, feasibility, acceptability, fidelity, cost).

*Context*: Any rehabilitation setting (acute care, inpatient rehabilitation, outpatient, community, home, telehealth)

*Study designs:* All empirical study designs including RCTs, non-randomized trials, cohort studies, case series (≥ 3 participants), case reports, mixed-methods studies, and published protocols.

### 2.3 Information sources

We searched the following databases from January 2010 to September 2025:

- PubMed/MEDLINE
- Cochrane Library (CENTRAL and Cochrane Reviews)
- PEDro (Physiotherapy Evidence Database)
- CINAHL (Nursing and Allied Health Literature)
- Web of Science Core Collection
- Google Scholar (first 200 results by relevance)

Additional sources included reference lists of included studies, clinical trial registries, and professional organization websites. A health sciences librarian peer-reviewed the strategy using PRESS criteria.

### 2.4 Search strategy

The search strategy combined three conceptual blocks using Boolean operators: Stroke block: “Stroke”[MeSH] OR “Hemiplegia”[MeSH] OR stroke OR strokes OR “cerebrovascular accident*” OR CVA OR hemipleg* OR hemipar*

Quadruped/Crawling block: quadruped* OR “all fours” OR “hands and knees” OR “four-point kneel*” OR “quadruped* position*” OR birddog OR “bird-dog” OR crawl* OR “bear crawl*” OR “kneel* train*”

Rehabilitation block: “Rehabilitation”[MeSH] OR “Exercise Therapy”[MeSH] OR “Physical Therapy Modalities”[MeSH] OR rehabilitat* OR physiotherap* OR “physical therap*” OR exercise* OR training.

Filters: Humans; Adults (≥ 19 years); English; 2010–2025

### 2.5 Study selection

The comprehensive database searches yielded 1,938 initial records. After removing 487 duplicates, 1,451 unique records remained for title/abstract screening. Two independent reviewers screened titles/abstracts after achieving 86% agreement on a 50-record calibration set. This initial screening excluded 1,289 records, leaving 162 records for full-text assessment.

**FIGURE 1:**
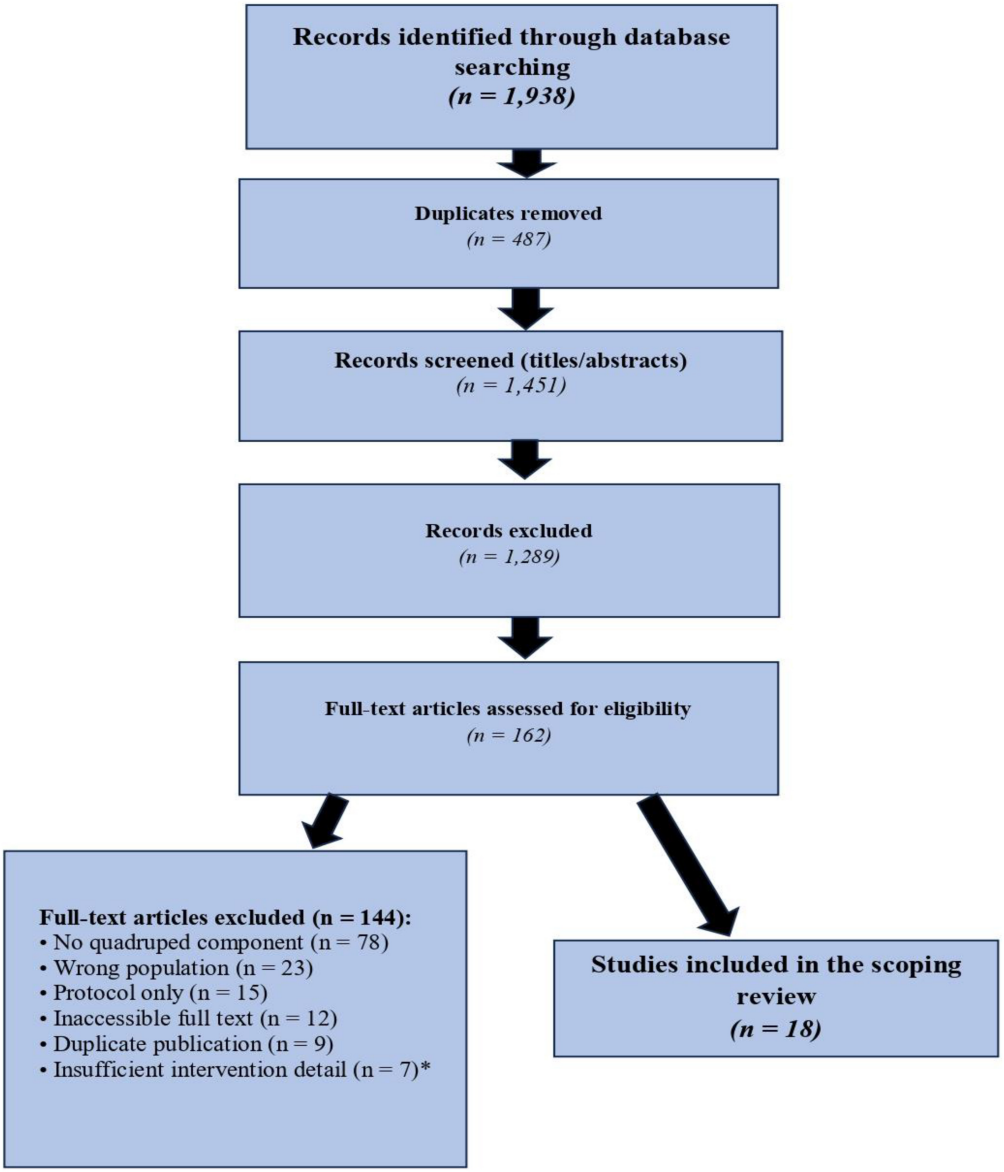
PRISMA-ScR flow diagram for study selection in the scoping review.

During full-text review, 144 articles were excluded for the following reasons: no explicit quadrupedal component (*n* = 78), wrong population (*n* = 23), protocol only without results (*n* = 15), unable to access full text (*n* = 12), duplicate publication (*n* = 9), and insufficient intervention detail (*n* = 7). The final corpus included 18 studies meeting all inclusion criteria (Figure 1).

The above figure shows records identified via database searches (*n* = 1,938), with duplicates removed (*n* = 487). The remaining records screened totaled 1,451, and 1,289 were excluded. Full texts assessed numbered 162, with 144 excluded for specific reasons. Ultimately, 18 studies were included in the final synthesis.

### 2.6 Data charting

A standardized data extraction form was piloted on five studies. Two reviewers independently extracted:

- Study characteristics (authors, year, country, design, funding)
- Participant characteristics (sample size, age, sex, stroke type, chronicity, severity)
- Intervention details (type, dosage, progression, equipment, setting)
- Outcomes and measures (ICF domains, assessment tools, time points)
- Implementation factors (recruitment, retention, adherence, barriers, facilitators)

### 2.7 Critical appraisal

While a formal risk-of-bias assessment is not required for scoping reviews, we mapped methodological quality indicators:

- For RCTs: PEDro scale scores
- For non-randomized studies: ROBINS-I assessment
- For case studies: CARE checklist compliance. Quality assessments were used for descriptive purposes only, not for study exclusion.

Two reviewers independently evaluated study quality using the PEDro, ROBINS-I, and CARE tools based on study design. They calibrated their assessments on a pilot sample of five studies. Any differences in scoring were settled through discussion and agreement, with a third reviewer arbitrating if needed.

### 2.8 Synthesis and analysis

We conducted descriptive synthesis with:

- Evidence mapping by ICF domains and intervention types
- Quantitative summary of participant characteristics and intervention parameters
- Thematic analysis of proposed mechanisms and implementation factors
- Gap analysis identifying understudied populations and missing outcome domains Where ≥ 3 studies reported comparable outcomes, we calculated standardized mean differences for illustrative purposes only.

### 2.9 Stakeholder consultation

Following initial synthesis, we conducted structured consultations with:

- Stroke survivors and caregivers (*n* = 8): acceptability, preferences, barriers
- Rehabilitation clinicians (*n* = 12): feasibility, training needs, implementation
- Researchers (*n* = 6): methodological recommendations, priority questions Qualitative feedback from these consultations was analyzed thematically to enhance the synthesis of implementation facilitators and barriers discussed in section 4.4 (Implementation Considerations). Full methodological details are available in Supplementary material 1.

## 3 Results

### 3.1 Study selection

The database search identified 1,938 records; 487 duplicates were removed. After screening 1,451 titles and abstracts and excluding 1,289, 162 full texts were assessed for eligibility. Of these, 144 were excluded for reasons such as the absence of a quadruped component (*n* = 78), incorrect population (*n* = 23), or protocol-only reports (*n* = 15). Ultimately, 18 studies met the inclusion criteria (Figure 1; Munn et al., 2023).

### 3.2 Study characteristics

Most stroke clinical studies were conducted in chronic outpatient settings (Chung et al., 2013; El-Nashar et al., 2019; Mahmood et al., 2022; Nadeem et al., 2024), with Zhang et al. (2024) focusing on subacute inpatients. A single-case report detailed improvements in a chronic stroke patient following a modified quadruped-derived program (Pascal et al., 2022). Stroke trials evaluated trunk control (TIS), balance (BBS), functional mobility (TUG), gait parameters (velocity, cadence, step length), and, in one study, quality of life (SS-QOL). Sample sizes ranged from 16 to 74 for outpatient studies and 67 for the inpatient RCT. Baseline participant characteristics and eligibility criteria are summarized in Supplementary Table 1.

Only the six stroke-specific studies were included in the quantitative analysis and outcome synthesis. The other studies offered contextual, mechanistic, or feasibility insights (see Figure 1). Outpatient studies (Chung et al., 2013; El-Nashar et al., 2019; Mahmood et al., 2022; Nadeem et al., 2024) addressed chronic stroke, whereas Zhang et al. (2024) focused on subacute inpatients. A single case report detailed improvements in chronic stroke following a modified quadruped- based program (Romanow et al., n.d.). Sample sizes varied from *n* = 16–74 for outpatient groups and *n* = 67 for inpatients. Overall, the trials evaluated trunk control (TIS), balance (BBS), and functional mobility (TUG, gait velocity, cadence), with one study also measuring quality of life (SS-QOL) (Mahmood et al., 2022).

Of the 18 included records, 6 were stroke-specific clinical studies (5 randomized controlled trials and 1 case report) and were used for the outcome synthesis; the remaining 12 provided mechanistic or translational context and were not pooled. Dose characteristics, supervision, and progression details for all included records (*n* = 18), stratified by evidence tier, are available in Supplementary Table 3.

### 3.3 Intervention typology and dosage

**FIGURE 2:**
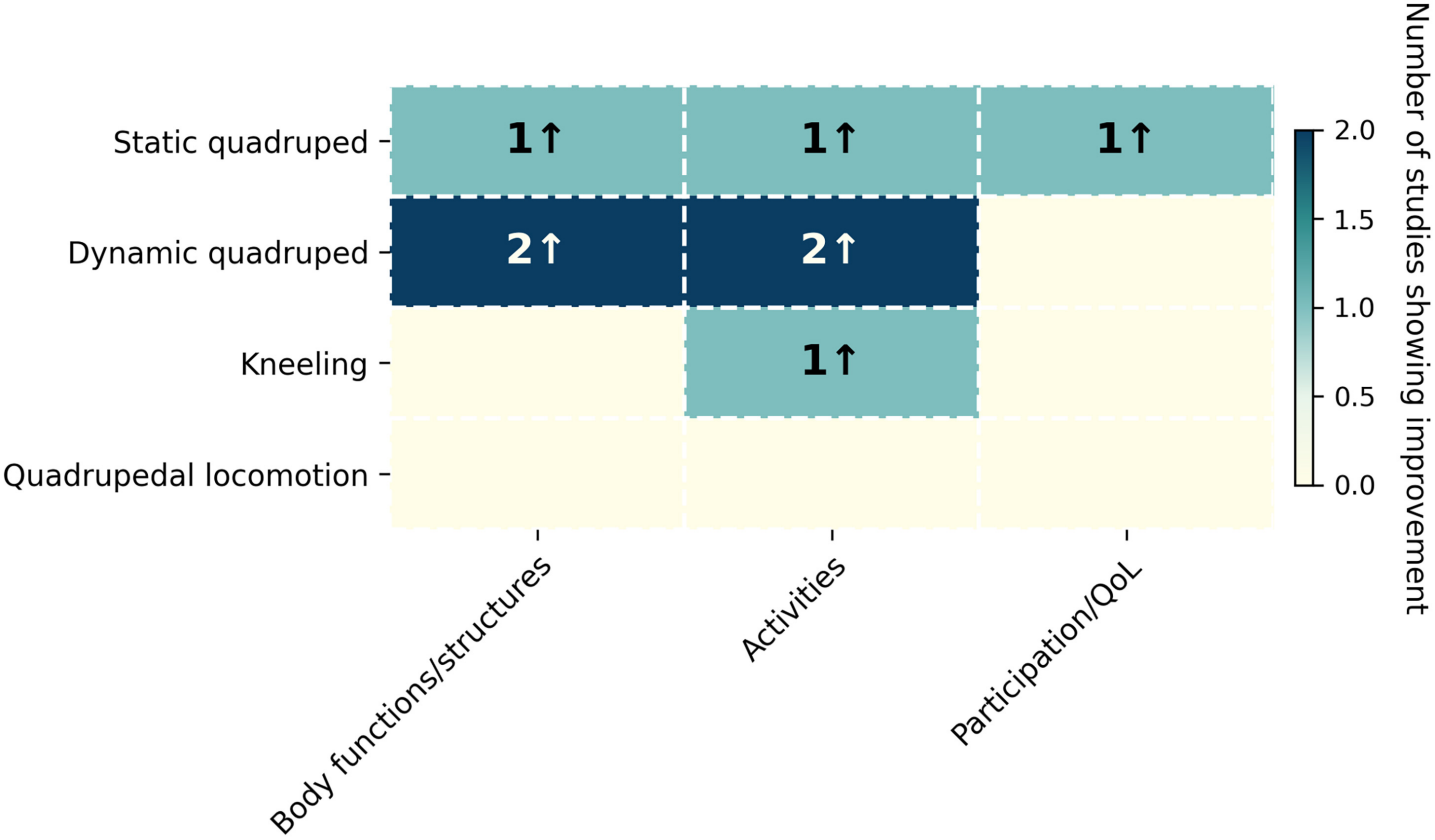
Evidence map of quadruped-derived training approaches in post-stroke rehabilitation. Rows represent intervention variants (static quadruped, dynamic quadruped, kneeling, and quadrupedal locomotion), and columns correspond to ICF domains (Body functions/structures, Activities, and Participation/QoL). Numbers indicate the count of stroke studies showing improvement (“↑”) within each domain.

Interventions clustered into four quadrupedal training (QT) variants (Figure 2 and Table 1):

**TABLE 1:** Quadruped-derived intervention typology, dose, supervision, and progression in stroke-specific clinical studies

Study	Stroke stage/setting	QT variant	QT minutes/ session	Total session minutes	Sessions/ week	Weeks	Weekly QT minutes	Total QT minutes	Supervision	Progression criteria	Comparator/role of QT
Chung et al. (2013)	Chronic; outpatient	Dynamic QT	NR	30 (QT) + 40 general training	3	4	NR	NR	Therapist-supervised (clinic)	Progression implied (bed → wedge → ball exercises); explicit rules NR	General exercise program; QT as adjunct
El-Nashar et al. (2019)	Chronic; outpatient	Dynamic QT	NR	30	3	6	NR	NR	Therapist-supervised (clinic)	Examples provided (bridging hold ∼10 s; reps), but formal criteria NR	Conventional PT; QT as adjunct
Mahmood et al. (2022)	Chronic ischemic; outpatient	Static + Dynamic QT	15	55 (40 conventional + 15 QT)	5	8	75	600	Therapist-supervised (clinic)	Progressive repetitions and hold duration described	Conventional therapy; QT as adjunct
Nadeem et al. (2024)	Chronic; outpatient	Dynamic QT (progressive core/QT)	15–20	30 (routine PT) + 15–20 QT	4	8	60–80	480–640	Therapist-supervised (clinic)	Stepwise progression (Stages I–III) with defined task advancement	Routine PT; QT as adjunct
Zhang et al. (2024)	Subacute; inpatient	Kneeling QT	30	30	6	4	180	720	Therapist-assisted (inpatient)	Progression via kneeling speed and trunk control; HR-guided intensity	Treadmill walking; QT as primary intervention
Pascal et al. (2022) (case)	Chronic; outpatient	Locomotor QT (crawling)	60	60	2	8	120	960	Therapist-supervised (clinic)	Progression from isolated movements to linked crawling sequences	None (single-case); QT as primary intervention

Weekly exposure was calculated as minutes per session × sessions per week. Dose values refer to the quadruped-derived component where reported; many protocols also included conventional rehabilitation. QT, quadruped training; NR, not reported; PT, physical therapy; HR, heart rate. QT = quadrupedal-derived training. QT-specific dose refers to time explicitly attributable to four-point, kneeling, or crawling-derived postures. When posture-specific exposure could not be isolated from broader intervention blocks, QT dose is reported as NR (not reported). Total session minutes include all co-interventions where applicable. Weekly QT minutes were calculated as QT minutes/session × sessions/week; total QT minutes were calculated as weekly QT minutes × program duration (weeks). Supervision refers to therapist-led delivery unless otherwise specified.

(a) Static QT: stationary four-point positions emphasizing isometric stabilization and multifidus activation (e.g., quadruped holds, core bracing).
(b) Dynamic QT: contralateral limb lifts and rhythmic patterns such as bird-dog, cat–camel, and weight-shift drills.
(c) Kneeling QT: tall or half-kneeling postures and transitional movements emphasizing proximal loading and hip-trunk alignment.
(d) Locomotor QT: sequences of crawling forward or backward with coordinated limb movements.

A comprehensive overview of dose parameters, supervision, and progression criteria across both stroke-specific and mechanistic records is presented in Supplementary Table 3.

Variants form a progression from static proximal control (static QT) → contralateral coordination (dynamic QT) → proximal loading in transitions (kneeling QT) → rhythmic interlimb locomotion (locomotor QT). Each variant progressively increases sensorimotor integration and trunk challenge, creating a spectrum from static control to dynamic interlimb coordination.

Dosing across trials varied from 360 to 720 minutes when quadruped work was the primary therapy (Chung et al., 2013; El-Nashar et al., 2019; Zhang et al., 2024) and approximately 480–640 min when used as an adjunct (Mahmood et al., 2022; Nadeem et al., 2024).

QT-specific dose interpretation is limited. While total intervention dose can be estimated across studies, isolating the QT-specific dose—such as time spent in true four-point, kneeling, or quadruped positions—was inconsistent. In many trials, QT was integrated into broader core stability or conventional therapy sessions, alongside exercises like bridging, curl-ups, range-of-motion exercises, or gait training. Consequently, reported dose values often reflect the designated experimental blocks rather than the actual posture-specific exposure. This limits the ability to directly infer dose–response relationships for QT itself. Nevertheless, a pattern emerges: lower-intensity, proximally focused QT used as an adjunct commonly correlates with improvements in trunk control and balance, while gait-related outcomes show more variability and may depend on higher doses or specific locomotor QT variants (e.g., crawling or kneeling sequences). When QT served as the primary intervention, balance improvements were noted even with shorter overall exposure, indicating that task relevance and posture are potentially as critical as total training time.

To enhance cross-study comparability, Table 1 provides a summary of minutes per session, sessions per week, program duration, estimated total exposure, supervision setting (clinic versus home), and reported progression criteria. When available, we extracted the dose attributable to quadruped-derived tasks; however, in several studies, QT content was integrated into broader core or conventional therapy blocks, and isolated time spent in quadruped-derived postures was not consistently reported.

### 3.4 Outcomes mapped to the ICF framework

Outcomes were mapped *a priori* to ICF domains to avoid post-hoc emphasis on responsive measures. Across ICF domains, signals were strongest for trunk and balance, followed by functional mobility, with some effects on gait and a single report of improvements in QoL. QT variants focusing on trunk control enhanced TIS (including dynamic sitting) and sagittal trunk mobility; cat–camel exercises added dynamic sitting without improvements in upper-limb motor function (El-Nashar et al., 2019). Dynamic contralateral training (e.g., bird-dog) reduced TUG times and increased gait speed and cadence compared with general exercise (Chung et al., 2013).

Kneeling exercises outperformed treadmill walking on BBS at weeks 2 and 4 and improved paretic step length, though no significant differences in FMA-LE were found between groups (Zhang et al., 2024). A static 4-point adjunct was associated with improved SS-QOL over conventional therapy alone (Mahmood et al., 2022). Overall, stroke trials showed benefits in at least one domain, with balance and trunk control being the most consistently improved; gait outcomes varied by intervention and dosage (Figure 2 and Table 1).

### 3.5 Feasibility and safety

Across all stroke RCTs, there were no reports of serious adverse events. The inpatient kneeling trial experienced two withdrawals due to mild low-back pain (Zhang et al., 2024); all other studies were completed without incident (Chung et al., 2013; El-Nashar et al., 2019; Mahmood et al., 2022; Nadeem et al., 2024). Adherence rates exceeded 90% where data were available. The required equipment was minimal, only mats and knee pads, supporting practical, low-cost scaling (Zhang et al., 2024). Routine comfort measures included knee pads/mats and neutral wrist wedges/blocks to prevent sustained wrist extension, which was emphasized during stakeholder consultation as crucial for promoting adoption and adherence.

### 3.6 Mechanistic and indirect evidence

**FIGURE 3:**
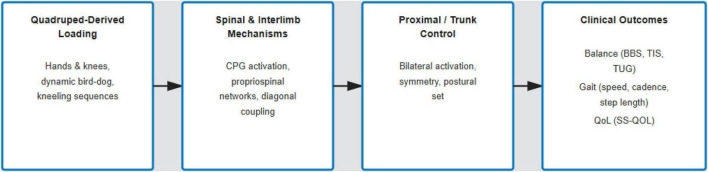
This logic model depicts a proposed neurophysiological pathway connecting quadruped-based training to functional recovery after stroke. It suggests that loading through quadruped exercises may activate spinal and interlimb coordination mechanisms, such as diagonal coupling and propriospinal pathways, while increasing demands on the proximal trunk. These effects could enhance postural symmetry and dynamic control, potentially leading to improvements in balance (e.g., BBS, TIS, TUG), gait parameters (speed, cadence, step length), and quality of life (SS-QOL). Most evidence supporting this pathway comes from non-stroke experimental and translational studies, so the model should be viewed as a plausible framework rather than a confirmed mechanism specific to stroke.

Mechanistic and translational evidence offers indirect support for the biological plausibility of QT, but it does not provide direct proof specific to stroke mechanisms. In healthy adults, muscle synergy analysis during hands-and-knees crawling revealed structured coordination patterns that may represent efficient control strategies for four-limb movement (Li et al., 2023). In an animal model, quadrupedal step training reactivated spinal interneuronal networks and enhanced locomotor outcomes compared to other training methods, supporting the idea that spinal and interlimb contributions are important for coordinated movement (Shah et al., 2013). A narrative review of infant crawling measurement also identified features relevant to rehabilitation, such as diagonal coupling and rhythmic coordination (Xiong et al., 2021). Overall, these findings suggest that quadruped-based loading and interlimb coupling could affect proximal control and downstream balance and mobility, as illustrated in Figure 3. They also highlight the need for mechanistic biomarkers in stroke-specific research.

Quadruped loading activates spinal and interlimb mechanisms, such as central pattern generators (CPGs) and propriospinal networks, which enhance bilateral trunk activation and postural symmetry, thereby improving balance (BBS, TIS, TUG), gait (speed, cadence, step length), and quality of life (SS-QOL). This rationale is supported by complementary inpatient evidence: a combination of core-strengthening and trunk NMES therapy improved K-BBS, PASS, and TIS scores over 3 weeks, outperforming either modality alone (Ko et al., 2016).

## 4 Discussion

These findings support the proposed QT mechanism, spinal/interlimb engagement leads to proximal/trunk control, which in turn results in improvements in balance and mobility, and encourage standardized dosing and direct comparisons to evaluate added benefit over traditional practices as illustrated in Figure 3.

### 4.1 Summary of evidence

This scoping review emphasizes a small yet consistent body of clinical studies on quadruped- based methods for stroke rehabilitation. The evidence from multiple RCTs and controlled trials shows improvements in balance measures, such as BBS and TUG, and trunk control, assessed by TIS and its subtests. Additionally, there are targeted improvements in gait metrics like velocity, cadence, and paretic step length, along with better quality of life reported in at least one outpatient trial. Notably, kneeling, a quadruped-derived technique emphasizing proximal loading, seems safe and practical during the early subacute phase and provides greater balance improvements than traditional treadmill-based therapy when the program duration is similar.

### 4.2 Why quadruped-derived training may work

Mechanistically, QT should be viewed as a network-level coordination task rather than just an isolated strengthening exercise. Four-limb loading increases afferent input and creates diagonal interlimb constraints, highlighting spinal and long propriospinal coordination across cervical–lumbar segments. This perspective aligns with both foundational and recent theories of interlimb coordination and locomotor control, where rhythmic coupling and distributed segmental interactions facilitate efficient whole-body movement (Guertin, 2013; Frigon, 2017). In this review, the strongest clinical evidence focuses on trunk control and balance, both likely affected by the need for proximal stabilization and by the symmetry constraints inherent in four-point and kneeling tasks.

Importantly, the mechanistic evidence in the included studies is mainly indirect: research on crawling synergy in healthy adults indicates structured coordination patterns during hands-and-knees locomotion (Li et al., 2023), and quadrupedal step-training after spinal cord injury shows that engaging all four limbs can reorganize interneuronal networks involved in coordinated stepping (Shah et al., 2013). These findings support plausibility but do not confirm stroke-specific neural mechanisms. Therefore, future QT research should combine clinical outcomes with mechanistic biomarkers (e.g., EMG synergy patterns, EEG/fNIRS signatures of bilateral engagement, and fMRI measures of interhemispheric functional connectivity; Marcantoni et al., 2024) to determine whether QT activates neural resources that are distinct from other task-specific rehabilitation methods.

Human neurophysiology reinforces this rationale. Synergy analyses during crawling show that the CNS reuses a compact set of shared synergies across various coordination modes, supporting economical control strategies even during complex, multilimbed movement (Li et al., 2023).

Interlimb-coupling research further documents strong bidirectional interactions between arms and legs, suggesting that arm drive can entrain or stabilize lower-limb timing, an effect relevant for post-stroke gait asymmetry (Arya and Pandian, 2014).

Animal models provide converging biological evidence: quadrupedal step training after spinal hemisection reorganizes rostrocaudal interneuronal networks, enhances hindlimb coordination, and reactivates locomotor circuits more effectively than isolated hindlimb training alone (Shah et al., 2013). Across species, QT appears well suited to stimulate bilateral proximal musculature, promote trunk symmetry, and leverage CPG-driven rhythmicity—mechanisms consistent with the improvements in TIS, BBS, TUG, and gait parameters observed across the included stroke studies.

Overall, the mechanistic logic summarized in Figure 3, proprioceptive loading → spinal/interlimb engagement → bilateral trunk activation → functional gains—offers a biologically coherent explanation for why QT shows early promise in post-stroke rehabilitation. The early promise of Quadrupedal Training (QT) in post-stroke rehabilitation is biologically explained by the coherent mechanistic logic illustrated in Figure 3. This model suggests a sequence leading to functional gains: proprioceptive loading → spinal/interlimb engagement → bilateral trunk activation.

### 4.3 Clinical implications

The emerging evidence suggests that quadruped-derived training (QT) may offer a practical and adaptable addition to contemporary neurorehabilitation, particularly for individuals with persistent deficits in trunk control and balance. Across both inpatient and outpatient settings, the most responsive participants were those in the subacute and chronic phases, who retained enough proximal strength and upper-limb weight-bearing tolerance to maintain a stable four-point position. These characteristics appear to create an optimal “entry window” for QT, allowing patients to benefit from the enriched proprioceptive environment of quadruped postures without imposing disproportionate mechanical strain.

In practice, QT requires remarkably little infrastructure. A firm mat, knee pads, and, when appropriate, neutral wrist wedges are typically sufficient. This minimalism is one of QT’s most substantial clinical advantages, enabling clinicians to introduce it seamlessly alongside existing therapeutic frameworks. The therapist’s primary responsibilities lie not in specialized equipment, but in movement quality: maintaining neutral alignment, preventing compensatory trunk rotation or excessive lumbar extension, and shaping the patient’s progression from static holds to more dynamic and transitional patterns. When integrated thoughtfully, QT can be dosed in short, focused blocks, often 15–30 min, and embedded within larger rehabilitation sessions without significant disruption of workflow.

A further implication arises from the variant-specific patterns observed in this review. Dynamic contralateral tasks tended to influence mobility outcomes, whereas kneeling-based interventions had more pronounced effects on balance. These distinctions suggest that QT may not function as a single homogeneous technique, but rather as a family of related strategies with subtly different targets and applications. For clinicians, this nuance provides an opportunity to tailor QT to patient profiles, prioritizing static control for those with reduced endurance or stability, and gradually introducing load-shifting or transitional kneeling patterns as postural confidence improves.

### 4.4 Implementation considerations

Implementing QT into everyday clinical settings involves considering various practical and perceptual factors. Feedback from stakeholders offered important insights: patients regularly described QT as accessible, engaging, and supportive of self-confidence, highlighting its straightforward structure and immediate postural feedback from four-point positions. These features may help overcome motivational issues commonly seen in long-term rehabilitation. The views shared by stroke survivors and caregivers (*n* = 8) closely aligned with the high adherence rates seen in clinical trials, which exceeded 90%.

At the same time, several barriers require proactive management. Discomfort in the wrists or knees can affect adherence, especially during early exposure. However, these issues are usually addressed with simple interventions, such as thicker mats, knee pads, wrist and hand braces, or wrist wedges, and rarely lead to the abandonment of quadruped practice entirely. Fear of falling during transitions to or from kneeling is another common concern. Clinicians can reduce this risk by providing close supervision during initial sessions and teaching graded transition techniques before allowing more autonomous practice.

Therapists’ confidence and familiarity also shape implementation. Many clinicians in stakeholder interviews reported that QT was not part of their formal training, making progression decisions less intuitive. This underscores the need for clear progression criteria and simple clinical algorithms to standardize the progression from static to dynamic tasks. Given its minimal equipment requirements, QT is well-suited to telerehabilitation, in which camera-based feedback and periodic in-person calibration could support hybrid care models. As rehabilitation increasingly moves toward home-based formats, QT’s simplicity and adaptability position it as a promising candidate for scalable deployment. Findings from structured stakeholder consultation with stroke survivors, clinicians, and researchers are summarized in Supplementary material 1 and directly inform the implementation themes discussed here.

### 4.5 Gaps and priorities for research

Despite encouraging results, the QT literature remains in an early developmental phase, with methodological, mechanistic, and translational gaps that must be addressed before widespread adoption can be recommended. One priority is the establishment of QT-specific outcome measures. Existing tools such as TIS, BBS, and TUG are appropriate for capturing downstream effects. Still, they do not assess quadruped-specific control demands such as endurance in four- point support, contralateral weight shifting, or controlled diagonal reach. Developing standardized tests in these domains would strengthen the ability to detect meaningful changes across QT variants.

A more fine-grained understanding of dose–response relationships is also needed. Total exposure across available trials varied widely, and only a few studies clearly documented progression criteria or fidelity measures. Future trials should incorporate transparent reporting standards (e.g., TIDieR) and specify how, when, and why exercises were progressed. Similarly, it remains unclear whether QT offers unique benefits at different stages of recovery or whether specific patient profiles, such as those with pronounced trunk asymmetry, derive disproportionate benefit. Well-controlled early-phase trials should therefore stratify by chronicity, baseline impairment, and compensatory movement patterns.

Finally, meaningful advancement will depend on linking mechanistic markers with clinical change. Techniques such as EEG, EMG synergy analysis, fNIRS, and task-based fMRI provide viable methods for quantifying interlimb coupling, cortical activation symmetry, and trunk–limb coordination during QT. Embedding these measures in future RCTs would help determine whether QT relies on spinal or cortical mechanisms distinct from those of other task-specific therapies, while similar treatment-parameter optimization questions have been raised in other post-stroke rehabilitation modalities (Wang et al., 2024). Longitudinal work is equally necessary to understand how QT influences walking endurance, community mobility, falls, and ADL performance.

### 4.6 Limitations of the evidence and of this review

As with many emerging rehabilitation approaches, the evidence base for QT is limited by small sample sizes, heterogeneous intervention designs, inconsistent reporting of progression or fidelity, and minimal long-term follow-up. Not all studies articulated clear clinical progression rules, and few captured mechanistic data alongside behavioral outcomes. This patchiness complicates efforts to compare results across trials or to extrapolate findings to broader clinical practice.

From a review methodology perspective, limiting searches to English publications and not performing a meta-analysis, as is common in scoping reviews, may introduce biases. Additionally, some included studies were mechanistic or involved non-stroke populations; while they enhance theoretical understanding, they do not replace stroke-specific clinical trials. Lastly, the stakeholder consultation used a convenience sample, which could lead to selection bias toward individuals more interested in rehabilitation and more favorable to QT. These limitations should be kept in mind when interpreting this synthesis and highlight the need for more rigorous experimental designs in future research.

## 5 Conclusion

The aim of this scoping review was to synthesize current evidence on the characteristics, mechanisms, and clinical applicability of quadrupedal-based training for adult post-stroke rehabilitation. The analysis shows that QT represents a promising, biologically grounded, and practical approach to post-stroke rehabilitation. Its signature elements, simultaneous upper- and lower-limb loading, diagonal interlimb coupling, and strong trunk engagement, align closely with known neurophysiological mechanisms that support postural symmetry and coordinated movement. The emerging clinical literature, though modest in size, consistently demonstrates improvements in trunk control and balance, with selective enhancements in gait when progression and dosage are optimized.

Given its low resource burden and adaptability across inpatient, outpatient, and home settings, QT offers an attractive complement to conventional therapy. Moving forward, well-designed trials that integrate mechanistic measures, standardized progression frameworks, and long-term functional endpoints will be essential. With these developments, QT has the potential to evolve from a promising adjunct to a structured, evidence-based intervention that can enrich neurorehabilitation practice across the continuum of stroke recovery.

## Data Availability

The original contributions presented in this study are included in the article/Supplementary material, further inquiries can be directed to the corresponding author.

## References

[B1] Arya K. N. Pandian S. (2014). Interlimb Neural Coupling: Implications for poststroke hemiparesis. *Ann. Phys. Rehabil. Med.* 57 696–713. 10.1016/j.rehab.2014.06.003 25262645

[B2] Bernhardt J. Hayward K. S. Kwakkel G. Ward N. S. Wolf S. L. Borschmann K. et al. (2024). Agreed definitions and a shared vision for new standards in stroke recovery research: The stroke recovery and rehabilitation roundtable taskforce. *Lancet Neurol.* 23 89–101. 10.1016/S1474-4422(23)00361-5

[B3] Buxton J. Daugherty M. Grubbs R. Isles M. Mulligan S. Plank E. et al. (2024). Comparison of muscle activation during quadrupedal movement training and traditional bodyweight exercises. *J. Bodyw. Mov. Ther.* 40, 2173–2178. 10.1016/j.jbmt.2024.11.002 39593581

[B4] Cole W. G. Vereijken B. Young J. W. Robinson S. R. Adolph K. E. (2019). Use it or lose it? Effects of age, experience, and disuse on crawling. *Dev. Psychobiol.* 61, 29–42. 10.1002/dev.21802 30447002 PMC6345180

[B5] Chung E.-J. Kim J.-H. Lee B.-H. (2013). The effects of core stabilization exercise on dynamic balance and gait function in stroke patients. *J. Phys. Ther. Sci.* 25 803–806. 10.1589/jpts.25.803 24259857 PMC3820398

[B6] Dietz V. (2011). Quadrupedal coordination of bipedal gait: Implications for movement disorders. *J. Neurol.* 258 1406–1412. 10.1007/s00415-011-6063-4 21553270

[B7] Dimitrijevic M. R. Gerasimenko Y. Pinter M. M. (2024). Evidence for a spinal central pattern generator in humans. *Ann. N. Y. Acad. Sci.* 1515 45–58. 10.1111/nyas.15234 9928325

[B8] El-Nashar H. ElWishy A. Helmy H. El-Rwainy R. (2019). Do core stability exercises improve upper limb function in chronic stroke patients? *Egypt. J. Neurol. Psychiatr. Neurosurg.* 55:38. 10.1186/s41983-019-0087-6

[B9] Feigin V. L. Stark B. A. Johnson C. O. Roth G. A. Bisignano C. Abady G. G. et al. (2021). Global, regional, and national burden of stroke and its risk factors, 1990–2019: A systematic analysis for the global burden of disease study 2019. *Lancet Neurol.* 20 795–820. 10.1016/S1474-4422(21)00252-0 34487721 PMC8443449

[B10] Fisher B. E. Wu A. D. Salem G. J. Song J. Lin C. H. Yip J. et al. (2024). Neural coupling during quadrupedal locomotion: Implications for post-stroke rehabilitation. *Neurorehabil. Neural Repair.* 38 189–201. 10.1177/15459683241228934

[B11] Frigon A. (2017). The neural control of interlimb coordination during mammalian locomotion. *J. Neurophysiol.* 117 2224–2241. 10.1152/jn.00978.2016 28298308 PMC5454475

[B12] Guertin P. A. (2013). Central pattern generator for locomotion: Anatomical, physiological, and pathophysiological considerations. *Front. Neurol.* 3:183. 10.3389/fneur.2012.00183 23403923 PMC3567435

[B13] Ko E. J. Chun M. H. Kim D. Y. Yi J. H. Kim W. Hong J. (2016). The additive effects of core muscle strengthening and trunk NMES on trunk balance in stroke patients. *Ann. Rehabil. Med.* 40 142–151. 10.5535/arm.2016.40.1.142 26949681 PMC4775748

[B14] Langhorne P. Coupar F. Pollock A. (2009). Motor recovery after stroke: A systematic review. *Lancet Neurol.* 8 741–754. 10.1016/S1474-4422(09)70150-4 19608100

[B15] Langhorne P. Wu O. Rodgers H. Ashburn A. Bernhardt J. (2017). A Very Early Rehabilitation Trial after stroke (AVERT): A Phase III, multicentre, randomised controlled trial. *Health Technol Assess.* 21, 1–120. 10.3310/hta21540 28967376 PMC5641820

[B16] Li C. Chen X. Zhang X. Chen X. Wu D. (2023). Muscle synergy analysis of eight inter-limb coordination modes during human hands-knees crawling movement. *Front. Neurosci.* 17:1135646. 10.3389/fnins.2023.1135646 37274209 PMC10235503

[B17] Lohse K. R. Lang C. E. Boyd L. A. (2014). Is more better? Using metadata to explore dose–response relationships in stroke rehabilitation. *Stroke* 45 2053–2058. 10.1161/STROKEAHA.114.004695 24867924 PMC4071164

[B18] MacKay-Lyons M. Billinger S. A. Eng J. J. Dromerick A. Giacomantonio N. Hafer- Macko C. et al. (2023). Aerobic exercise recommendations to optimize best practices in care after stroke: AEROBICS 2023 update. *Phys. Ther.* 103:zad078. 10.1093/ptj/pzad078 31596465 PMC8204880

[B19] Mahmood W. Ahmed Burq H. S. I. Ehsan S. Sagheer B. Mahmood T. (2022). Effect of core stabilizationexercises in addit ion to conventional therapy in improving trunk mobility, function, ambulation and quality of life in stroke patients: A randomized controlled trial. *BMC Sports Sci. Med. Rehabil.* 14:62. 10.1186/s13102-022-00452-y 35395819 PMC8991663

[B20] Marcantoni I. Piccolantonio G. Ghoushi M. Valenti M. Reversi L. Mariotti F. et al. (2024). Interhemispheric functional connectivity: An FMRI study in callosotomized patients. *Front. Hum. Neurosci.* 18:1363098. 10.3389/fnhum.2024.1363098 38812473 PMC11133720

[B21] Mitchell L. Ziviani J. Oftedal S. Boyd R. (2012). The effect of virtual reality interventions on physical activity in children and adolescents with early brain injuries including cerebral palsy. *Dev. Med. Child Neurol.* 54 667–671. 10.1111/j.1469-8749.2011.04199.x 22283557

[B22] Munn Z. Pollock D. Khalil H. Alexander L. McInerney P. Godfrey C. M. et al. (2023). What are scoping reviews? Providing a formal definition of scoping reviews as a type of evidence synthesis. *JBI Evid. Synth.* 21 520–532. 10.11124/JBIES-22-00123 35249995

[B23] Nadeem I. Butt S. K. Mubeen I. Razzaq A. (2024). Effects of core muscles strengthening exercises with routine physical therapy on trunk balance in stroke patients: A randomized controlled trial. *J. Pak. Med. Assoc.* 74 848–851. 10.47391/JPMA.9660 38783428

[B24] Pascal E. Morgan C. Singh N. (2022). *Modified Quadruped-Based Exercise to Improve Function in a Chronic Stroke Patient: A Case Study.* Dallas, PA: Misericordia University.

[B25] Prosser L. A. Lee S. C. K. VanSant A. F. Barbe M. F. Lauer R. T. (2010). Trunk and hip muscle activation patterns are different during walking in young children with and without cerebral palsy. *Phys. Ther.* 90, 986–997. 10.2522/ptj.20090161 20430948 PMC2897131

[B26] Shah P. K. Garcia-Alias G. Choe J. Gad P. Gerasimenko Y. Tillakaratne N. et al. (2013). Use of quadrupedal step training to re-engage spinal interneuronal networks and improve locomotor function after spinal cord injury. *Brain* 136 3362–3377. 10.1093/brain/awt265 24103912 PMC3808689

[B27] Stinear C. M. Lang C. E. Zeiler S. Byblow W. D. (2020). Advances and challenges in stroke rehabilitation. *Lancet Neurol.* 19 348–360. 10.1016/S1474-4422(19)30415-6 32004440

[B28] Tricco A. C. Lillie E. Zarin W. O’Brien K. K. Colquhoun H. Levac D. et al. (2018). PRISMA extension for scoping reviews (PRISMA-ScR): Checklist and explanation. *Ann. Intern. Med.* 169 467–473. 10.7326/M18-0850 30178033

[B29] Wang C. Nie P. Wang P. Wang Y. Zang Y. Zhang Y. (2024). The Therapeutic effect of transcranial magnetic stimulation on post-stroke aphasia and the optimal treatment parameters: A meta- analysis. *Arch. Phys. Med. Rehabil.* 105 1388–1398. 10.1016/j.apmr.2023.11.006 37984539

[B30] Ward N. S. Brander F. Kelly K. (2019). Intensive upper limb neurorehabilitation in chronic stroke: Outcomes from the Queen Square programme. *J. Neurol. Neurosurg. Psychiatry* 90 498–506. 10.1136/jnnp-2018-319954 30770457

[B31] Winters C. Kwakkel G. van Wegen E. E. H. Nijland R. H. M. Veerbeek J. M. Meskers C. G. M. (2018). Moving stroke rehabilitation forward: The need to change research. *NeuroRehabilitation* 43, 19–30. 10.3233/NRE-172393 30056434

[B32] Xiong Q. L. Wu X. Y. Liu Y. Zhang C. X. Hou W. S. (2021). Measurement and analysis of human infant crawling for rehabilitation: A narrative review. *Front. Neurol.* 12:731374. 10.3389/fneur.2021.731374 34707557 PMC8544808

[B33] Zehr E. P. Duysens J. (2004). Regulation of arm and leg movement during human locomotion. *Neuroscientist* 10 347–361. 10.1177/1073858404264680 15271262

[B34] Zhang L. Chen M. Li X. Wang Y. Liu J. (2024). The effect of kneeling training on balance and functional mobility in stroke patients: A randomized controlled trial. *BMC Sports Sci. Med. Rehabil.* 16:163. 10.1186/s13102-024-00953-y 39095858 PMC11295609

