# Supplementary material for: Quadrupedal training approaches in post-stroke rehabilitation: a scoping review of evidence, mechanisms, and clinical applications
Source: Front Syst Neurosci. 2026 Apr 8;20:1773330. doi: 10.3389/fnsys.2026.1773330 (PMC13099906; doi:10.3389/fnsys.2026.1773330)
Supplement: Supplementary file 1 [file Data_Sheet_1.pdf]

Supplementary Material S1

Stakeholder Consultation and Implementation Insights for Quadrupedal-Derived Training After Stroke

Purpose of This Supplement

This supplementary document provides transparent documentation of the **stakeholder consultation process** undertaken as part of the scoping review *"Quadrupedal Training Approaches in Post-Stroke Rehabilitation: A Scoping Review of Evidence, Mechanisms, and Clinical Applications."* This supplement aims to demonstrate methodological rigor and to substantiate how stakeholder input informed the interpretation of feasibility, acceptability, and implementation considerations reported in **Section 4.4 (Implementation Considerations)** of the main manuscript.

Consistent with JBI guidance for scoping reviews, stakeholder consultation was conducted to enhance clinical relevance, contextual validity, and translational applicability of the synthesized evidence.

1. Methods

1.1 Stakeholder Groups and Recruitment

Following completion of the initial evidence synthesis and extraction tables, structured consultations were conducted with three stakeholder groups to enhance clinical relevance and translational validity, consistent with JBI guidance for scoping reviews.

- **Stroke survivors and caregivers** *(n = 8)*
  Adults with lived experience of stroke rehabilitation, representing both subacute and chronic stages of recovery. Caregivers included informal family members involved in daily mobility and exercise support.
- **Rehabilitation clinicians** *(n = 12)*
  Licensed professionals actively involved in stroke rehabilitation, including physiotherapists, occupational therapists, and exercise-based neurorehabilitation practitioners across inpatient and outpatient settings.
- **Researchers** *(n = 6)*
  Researchers with expertise in neurorehabilitation, motor control, neurophysiology, and clinical trial methodology.

Participants were recruited through professional networks, clinical contacts, and prior engagement with stroke rehabilitation programs. Participation was voluntary, and no identifying or sensitive personal information was collected.

1.2 Consultation Format and Content

Consultations were conducted using semi-structured formats (individual interviews or small-group discussions). Guiding questions focused on:

- Perceived **acceptability** of quadrupedal-derived training (QT)
- Practical **barriers and facilitators** to implementation
- **Training needs** and decision-making challenges for clinicians
- Perceived **relevance of outcomes** used in current studies
- Recommendations regarding **dosing, progression, and safety**
- Methodological priorities for future clinical trials

Sessions were documented using structured field notes. The objective was to capture recurring implementation-relevant themes rather than to conduct in-depth qualitative phenomenological analysis.

1.3 Analytic Approach

Qualitative feedback was analyzed using **thematic content analysis**. Two reviewers independently reviewed consultation notes and coded responses into preliminary categories. Codes were iteratively refined and grouped into higher-order themes through discussion and consensus. Themes were compared across stakeholder groups to identify convergence and divergence.

The resulting themes were used to:

- Contextualize feasibility and adherence findings in the Results section
- Inform the implementation-focused synthesis in Section 4.4 of the Discussion
- Identify translational gaps and research priorities outlined in Section 4.5

---

2. Results: Stakeholder-Derived Themes

2.1 Acceptability and User Experience

Across consultations, **stroke survivors and caregivers** consistently described quadrupedal-derived exercises as intuitive and less threatening than upright balance tasks. Many

participants emphasized that training close to the ground reduced fear of falling and provided immediate sensory feedback, particularly regarding trunk alignment and weight distribution.

Several caregivers noted that QT exercises were easier to support at home compared with standing balance or gait tasks, as assistance could be provided without complex guarding techniques.

Key acceptability facilitators included:

- A perceived sense of safety due to a low center of mass
- Clear task structure and repetition
- Early subjective improvements in trunk awareness and postural confidence

These perceptions aligned closely with the high adherence rates (>90%) reported in the stroke-specific trials included in the review.

2.2 Practical Barriers and Risk Management

Stakeholders identified several recurring barriers to QT implementation. **Physical discomfort** was the most frequently reported issue, particularly wrist extension loading during sustained four-point positions and knee pressure during kneeling-based tasks. Stroke survivors also described fatigue during early sessions, especially when static holds were prolonged without sufficient rest intervals.

Fear of falling was primarily associated with transitions between low quadruped, kneeling, and standing. Clinicians emphasized that this concern diminished when progression was gradual and when transitions were explicitly trained rather than assumed.

Importantly, stakeholders viewed these barriers as **modifiable**, not exclusionary. Simple adaptations—such as thicker mats, knee pads, neutral wrist wedges, shorter initial bouts, and therapist-supported transitions—were widely considered sufficient to maintain participation.

2.3 Facilitators and Implementation Strategies

Multiple facilitators supporting successful QT integration were identified across stakeholder groups. Clinicians emphasized that QT aligned well with existing neurorehabilitation principles and could be inserted into sessions without restructuring treatment plans.

Commonly cited facilitators included:

- Use of **knee pads, thick mats, and neutral wrist supports**
- Gradual progression from static to dynamic and transitional tasks
- External visual feedback (mirrors or video) to reinforce symmetry

- Short, frequent practice blocks rather than prolonged sessions

Clinicians also highlighted that QT allowed for clearer observation of compensatory strategies, enabling real-time correction of trunk rotation or asymmetrical loading.

2.4 Training Needs for Clinicians

Rehabilitation clinicians consistently reported that quadrupedal-derived training was **underrepresented in formal education and certification programs**. As a result, uncertainty often existed regarding:

- When to progress from static to dynamic QT
- How to individualize progression for asymmetrical trunk control
- How to integrate QT with upright gait and functional task training

Stakeholders recommended the development of simple progression frameworks, visual decision aids, and example protocols with explicit entry and exit criteria.

2.5 Research Priorities Identified by Stakeholders

Stakeholder input strongly converged on several research priorities:

- Development of **QT-specific outcome measures** capturing endurance, symmetry, and weight-shifting capacity
- Dose–response studies with clearly reported total exposure and progression logic
- Integration of **neurophysiological measures** (e.g., EMG, EEG, fNIRS) to link mechanistic hypotheses to clinical outcomes
- Longer-term follow-up examining carryover to walking, activities of daily living, and community participation

---

2.2 Practical Barriers

Across stakeholder groups, several recurrent barriers were identified:

- **Physical discomfort**: wrist extension load, knee pressure during kneeling
- **Fear of falling** during transitions between quadruped, kneeling, and standing
- **Fatigue** during sustained four-point positions
- **Clinician uncertainty** regarding progression and dosing

Stakeholders emphasized that these barriers were typically **modifiable** rather than exclusionary.

2.3 Facilitators and Implementation Strategies

Stakeholders identified multiple facilitators that supported successful implementation:

- Use of **knee pads, thick mats, and neutral wrist wedges** to manage joint loading
- Gradual progression from static to dynamic and transitional tasks
- Clear verbal cueing and external visual feedback (e.g., mirrors, video)
- Short, frequent training blocks rather than prolonged sessions

Clinicians noted that QT integrated well into existing therapy sessions and did not require specialized equipment.

2.4 Training Needs for Clinicians

Clinicians reported that quadrupedal-derived training was **underrepresented in formal education**, leading to uncertainty around:

- When to progress from static to dynamic tasks
- How to individualize QT for patients with asymmetrical trunk control
- How to integrate QT with gait and upright task training

Stakeholders suggested the need for:

- Simple progression frameworks
- Visual decision aids or flowcharts
- Example protocols with defined entry and exit criteria

2.5 Research Priorities Identified by Stakeholders

Researchers and clinicians highlighted several priorities for future investigation:

- Development of **quadruped-specific outcome measures** (e.g., endurance or weight-shift control in four-point)
- Dose–response studies with clearly reported total exposure and progression criteria
- Integration of **neurophysiological measures** (EMG, EEG, fNIRS) to link mechanism to outcome

- Longer-term follow-up assessing carryover to walking, ADLs, and community participation

---

3. Integration With Main Manuscript

Themes from this consultation directly informed:

- **Section 3.5 (Feasibility and Safety)**: interpretation of adherence and adverse event reporting
- **Section 4.4 (Implementation Considerations)**: barriers, facilitators, and clinical strategies
- **Section 4.5 (Gaps and Priorities for Research)**: alignment with real-world needs

This supplement therefore serves as transparent evidence that stakeholder input was systematically collected, analyzed, and integrated into the review.

---

4. Integration With Main Manuscript and Conclusions

4.1 Integration With Main Manuscript

Themes derived from stakeholder consultation directly informed multiple sections of the main manuscript:

- **Section 3.5 (Feasibility and Safety):** interpretation of adherence rates and management of joint discomfort
- **Section 4.4 (Implementation Considerations):** identification of barriers, facilitators, and practical strategies
- **Section 4.5 (Gaps and Priorities for Research):** alignment of future research directions with real-world needs

This explicit integration ensures that implementation considerations were grounded not only in trial-level data but also in lived experience and clinical expertise.

4.2 Conclusions

This supplementary analysis demonstrates that stakeholder consultation added meaningful depth to the scoping review by clarifying acceptability, feasibility, and translational relevance of quadrupedal-derived training after stroke. While barriers related to joint loading, fatigue, and

clinician confidence were identified, stakeholders consistently emphasized that these challenges were addressable through simple adaptations and clearer progression guidance.

The convergence between stakeholder perceptions and trial-level adherence supports the scalability of QT across inpatient, outpatient, and home-based contexts. Moreover, stakeholder-identified research priorities closely mirrored gaps identified in the formal evidence synthesis, reinforcing the validity of the proposed future research agenda.

Together, these findings substantiate the inclusion of stakeholder consultation as a valuable component of scoping reviews addressing complex rehabilitation interventions.
