# Supplementary material for: Quadrupedal training approaches in post-stroke rehabilitation: a scoping review of evidence, mechanisms, and clinical applications
Source: Front Syst Neurosci. 2026 Apr 8;20:1773330. doi: 10.3389/fnsys.2026.1773330 (PMC13099906; doi:10.3389/fnsys.2026.1773330)
Supplement: Supplementary file 2 [file Data_Sheet_2.pdf]

Paper	Study design	Participant age, sex and count	Setting (acute/inpatient/outpatient/home/tele)	Stroke profile (type, chronicity/time-since, lesion side/location)	Baseline severity (primary scale, e.g., NIHSS/FMA/BBS)	Intervention label + quadruped variant (static/dynamic/locomotion/kneeling) & main tasks	Dose (session min, sessions/week, program weeks, total minutes)	Comparator & co-interventions (if any)	Intensity metric (RPE, %HRR, etc.)	Outcomes mapped to ICF + timepoints (baseline/post/follow-up)	Key findings (direction & magnitude; note stats if reported)	Safety & feasibility: adverse events, adherence, retention	Mechanistic measures/rationale (EMG/EEG/TMS/fMRI; interlimb coupling/IHI/CPG)	Fidelity (checks/deviations)	Acceptability (satisfaction/qual quotes)	Cost/resource use (therapist time, equipment)	Funding/COI/Trial registration
The Additive Effects of Core Muscle Strengthening and Trunk NMES on Trunk Balance in Stroke Patients<br>Min Ho +6<br>Annals of Rehabilitation Medicine<br>arm-40-142.pdf<br>2016 ·<br>25 citations	Randomized, prospective cohort study, partially blinded (evaluators blinded), single-site, non-controlled (no placebo or control group without intervention), parallel design	- CMS group: 8 males, 2 females, median age 59.5 years<br>- tNMES group: 4 males, 6 females, median age 65.5 years<br>- Combination group: 7 males, 3 females, median age 58.5 years	inpatient	- Type: Acute and subacute stroke; cerebral infarction (26 patients), cerebral hemorrhage (4 patients)<br>- Chronicity/Time-since: Within 1 month of onset; specific days post-stroke: CMS group (12 days), tNMES group (8.5 days), Combination group (11 days)<br>- Lesion side/location: Side of weakness - CMS group (5:5), tNMES group (6:4), Combination group (4:6)	Not mentioned (the paper does not provide information on baseline severity using scales like NIHSS or FMA)	- Intervention label: Core Muscle Strengthening (CMS)<br>- Quadruped variant: Static (supine, prone, lateral positions)<br>- Main tasks: Trunk muscle strengthening, selective movements, coordination	- Session minutes: 20<br>- Sessions/week: 3<br>- Program weeks: 3<br>- Total minutes: 180	- Comparator: CMS group vs. tNMES group vs. Combination group (CMS + tNMES)<br>- Co-interventions: Standard rehabilitation program for all groups	Intensity metric: 30-70 mA electrical stimulation intensity; subjective maximum intensity without pain or tiredness	- K-BBS (trunk balance): baseline, post (3 weeks)<br>- PASS (postural assessment): baseline, post (3 weeks)<br>- TIS (trunk impairment): baseline, post (3 weeks)<br>- K-MBI (ADL): baseline, post (3 weeks)	- The combination group showed more improvements in K-BBS and dynamic sitting balance of TIS compared to CMS and tNMES groups.<br>- All groups improved in K-BBS, PASS, TIS, and K-MBI, but the combination group had more significant improvements.<br>- Additive effect of CMS and tNMES on trunk balance recovery was observed.<br>- Improvements in K-MBI were not correlated with improvements in K-BBS, PASS, or static sitting balance subscale of TIS.	- Adverse events: None reported due to treatment.<br>- Adherence: All 30 patients who completed the study were included in the analysis.<br>- Retention: Four patients dropped out due to early discharge or poor medical condition.	- Mechanistic measures/rationale: Core muscle strengthening (CMS) for muscle strengthening and coordination; Trunk neuromuscular electrical stimulation (tNMES) to strengthen trunk extension and increase cortical excitability.<br>- Theoretical basis: CMS aims to improve muscle function and control; tNMES increases cortical excitability and induces long-term plasticity in motor maps.<br>- Limitation: No use of surface electromyography to assess trunk muscle activity.	- tNMES: 4 channel electrodes attached to thoracic erector spinae and lumbar erector spinae; electrical stimulation at 30-70 mA intensity, 250 ms pulse width, and 35 Hz frequency.<br>- CMS: Trunk muscle strengthening, selective movements, and coordination exercises in supine, prone, and lateral positions.<br>- Standardized assessment tools: K-BBS, PASS, TIS, K-MBI.<br>- Random assignment of patients and therapists; some therapists blinded to treatment allocation.<br>- Detailed baseline characteristics and outcome measures tracked.	Not mentioned (the paper does not provide specific information on patient satisfaction or acceptability)	The paper mentions the use of therapist time for 20-minute sessions, 3 times a week for 3 weeks, and the use of specific equipment (Novastim CU-FS1) for tNMES, but does not provide detailed cost information or additional resource use beyond these descriptions.	Not mentioned (no information on funding, COI, or trial registration is included in the paper)
Central pattern generator for locomotion: anatomical, physiological, and pathophysiological considerations<br>Pierre A Guertin +2<br>Front. Neur.<br>fneur_03_00183_1.pdf<br>2013 ·<br>224 citations	Systematic review	Not mentioned (the paper is a review and does not provide specific participant demographics)	Not applicable (the paper is a review and does not specify a setting)	Not mentioned (the paper does not discuss stroke or provide a stroke profile)	Not mentioned (the paper does not provide baseline severity scores or mention specific scales like NIHSS/FMA/BBS)	- Intervention label: Restoration of bipedal walking in UTS patients<br>- Quadruped variant: Quadrupedal gait<br>- Main tasks: Restoration of normal CPG coupling and activity, promotion of bipedal walking	Not mentioned (the paper does not provide specific dosing information for the interventions discussed)	Not mentioned (the paper does not specify a comparator or co-intervention in the context of a controlled study)	Not mentioned (the paper does not discuss intensity metrics like RPE or %HRR)	Not mentioned (the paper does not provide specific outcomes mapped to ICF or timepoints)	- The CPG for locomotion can self-produce rhythmic movements without descending or peripheral inputs.<br>- Plasticity changes in CPG elements contribute to pathophysiological conditions affecting locomotion.<br>- The CPG is localized in the lumbar area of the spinal cord.<br>- Specific receptors (NMDA, 5-HT1, 5-HT7, 5-HT2A, D1) are involved in CPG activation.<br>- Simultaneous activation of these receptors induces full locomotor effects.<br>- Epidural electrical stimulation triggers stepping-like movements in SCI patients.<br>- The spinal locomotor network undergoes adaptations and plasticity changes post-SCI.<br>- Immediate early gene expression changes occur post-trauma.<br>- Nitric oxide synthase expression increases	Not mentioned (the paper does not provide specific information on adverse events, adherence, or retention rates)	- Mechanistic measures: Pharmacological manipulations using receptor ligands (agonists and antagonists) to understand CPG organization.<br>- Rationale: Identification of specific receptors (NMDA, 5-HT1, 5-HT7, 5-HT2A, D1) involved in CPG activation using selective antagonists and genetically manipulated animals.<br>- Interlimb coupling/IHI/CPG: CPG for locomotion is localized in the lumbar area, controlling basic motor commands for ambulation; flexibility and adaptability of the CPG are key for therapeutic approaches.	The CPG for locomotion is flexible and adaptable, showing fidelity in its ability to adjust and maintain rhythmic motor behaviors despite changes or injuries. It undergoes adaptations and plasticity changes following spinal cord injuries, indicating a form of fidelity in maintaining locomotor functions. Regulatory changes and plasticity in response to injury suggest a mechanism for maintaining fidelity in locomotor patterns. The return to near-normal conditions after certain interventions indicates a level of fidelity in the CPG's ability to recover and maintain normal function.	The author declares financial competing interests in Nordic Life Science Pipeline, which is a standard disclosure but may affect perceptions of acceptability or satisfaction.	The paper does not provide specific details on the cost or resource use of therapies such as BWSTT or pharmacological aids. However, it implies that these interventions require significant resources in terms of therapist time and equipment.	- Funding: Not mentioned<br>- COI: The author declares financial competing interests in Nordic Life Science Pipeline<br>- Trial registration: Not mentioned

											post-injury.<br>- Pharmacological aids like adrenergic α1 agonists increase leg extensor activity.						
Effect of crawling training on the cognitive function of children with cerebral palsy<br>Yan Bai +1<br>International journal of rehabilitation research. Internationale Zeitschrift fur Rehabilitationsforschung. Revue internationale de recherches de readaptation<br>effect_of_crawling_training_on_the_cognitive.11.pdf<br>2022<br>3 citations	- Retrospective case-control study<br>- Controlled<br>- Non-randomized<br>- Single-center	- Experimental Group: 26 boys, 10 girls, aged 1.24 ± 0.36 years, total count = 36<br>- Control Group: 24 boys, 8 girls, aged 1.25 ± 0.33 years, total count = 32	inpatient/outpatient (hospital setting)	Not mentioned (the paper focuses on cerebral palsy, not stroke)	GMFCS ratings: Experimental group - Level I = 19, Level II = 10, Level III = 7; Control group - Level I = 16, Level II = 9, Level III = 7	Crawling training; Locomotion; Crawling	Not mentioned (the paper does not specify the session duration, frequency per week, or total minutes of the program)	- Comparator: Normal movement training<br>- Co-interventions (for control group): Trunk control, sitting and lying postural changes, massage, stimulation of limbs, passive stretching of limbs	Not mentioned (the paper does not provide any information on intensity metrics such as RPE or %HRR)	- Crawling function: Baseline (Experimental: 20.93±3.85, Control: 20.97±3.79), Post-treatment (Experimental: 32.02±4.33, Control: 27.11±4.05)<br>- Cognitive and language functions: Baseline (not specified), Post-treatment (Experimental: significantly increased, Control: significantly increased)<br>- Intelligence quotient (IQ): Baseline (Experimental: 60.02±6.46, Control: 60.11±6.38), Post-treatment (Experimental: significantly increased, Control: significantly increased)<br>- Development quotient (DQ): Baseline (Experimental: 52.44±6.85, Control: 52.51±6.79), Post-treatment (Experimental: significantly increased, Control: significantly increased)<br>- Attention factor: Baseline (not specified), Post-treatment (Experimental: significantly increased, Control: significantly increased)<br>- Total effective rate: Baseline (not applicable), Post-treatment (Experimental: 94.44%, Control: 71.87%)	- Total treatment efficiency: Experimental group (94.44%) vs. Control group (71.87%), P = 0.012.<br>- Crawling function: Increased significantly in both groups; Experimental group (t = 11.18; P < 0.001) vs. Control group (t = 6.46; P < 0.001).<br>- Cognitive and language functions: Increased significantly in the experimental group; Experimental group > Control group, P < 0.05.<br>- IQ, DQ, and attention factor: Increased significantly in the experimental group; Experimental group > Control group, P < 0.05.	Not mentioned (the paper does not provide specific information on adverse events, adherence, or retention rates)	Not mentioned (the paper does not include any mechanistic measures such as EMG/EEG/TMS/fMRI or interlimb coupling/IHI/CPG)	Not mentioned (the paper does not provide information on fidelity or adherence to the treatment protocol)	- The total treatment efficiency of the experimental group was significantly higher than that of the control group (94.44 vs. 71.87%, P = 0.012).<br>- Crawling training has good clinical efficacy for patients with cerebral palsy.<br>- Crawling training is significantly effective in the treatment of children with cerebral palsy and notably improves crawling and cognitive functions.<br>- Accordingly, it is worthy of clinical promotion.	Not mentioned (the paper does not provide information on cost or resource use such as therapist time or equipment)	Not mentioned (no information on funding, COI, or trial registration is included in the paper)

Effect of crawling training on the cognitive function of children with cerebral palsy<br>Yan Bai +1<br>International journal of rehabilitation research. Internationale Zeitschrift fur Rehabilitationsforschung. Revue internationale de recherches de readaptation<br>effect_of_crawling_training_on_the_cognitive11.pdf<br>2022 ·<br>3 citations	- Retrospective case-control study<br>- Controlled<br>- Non-randomized<br>- Single-center	- Experimental Group: 26 boys, 10 girls, mean age = 1.24 years, total = 36<br>- Control Group: 24 boys, 8 girls, mean age = 1.25 years, total = 32	inpatient/outpatient (hospital setting)	Not mentioned (the paper does not discuss stroke or provide any stroke profile information)	GMFCS ratings: Experimental group - Level I = 19, Level II = 10, Level III = 7; Control group - Level I = 16, Level II = 9, Level III = 7	Crawling training; Locomotion; Crawling	Not mentioned (the paper does not specify the session duration, frequency per week, or total minutes of the program)	- Comparator: Normal movement training<br>- Co-interventions (for control group): Trunk control, sitting and lying postural changes, massage, stimulation of limbs, passive stretching of limbs	Not mentioned (the paper does not provide any information on intensity metrics such as RPE or %HRR)	- Crawling function: Baseline (Experimental: 20.93±3.85, Control: 20.97±3.79), Post-treatment (Experimental: 32.02±4.33, Control: 27.11±4.05)<br>- Cognitive and language functions: Baseline (not specified), Post-treatment (Experimental: significantly increased, Control: significantly increased)<br>- Intelligence quotient (IQ): Baseline (Experimental: 60.02±6.46, Control: 60.11±6.38), Post-treatment (Experimental: significantly increased, Control: significantly increased)<br>- Development quotient (DQ): Baseline (Experimental: 52.44±6.85, Control: 52.51±6.79), Post-treatment (Experimental: significantly increased, Control: significantly increased)<br>- Attention factor: Baseline (not specified), Post-treatment (Experimental: significantly increased, Control: significantly increased)<br>- Total effective rate: Baseline (not applicable), Post-treatment (Experimental: 94.44%, Control: 71.87%)	- Total treatment efficiency: Experimental group (94.44%) vs. Control group (71.87%), P = 0.012.<br>- Significant improvements in crawling function, cognitive and language functions, intelligence quotient, development quotient, and attention factor in both groups.<br>- Experimental group had significantly higher scores than the control group after treatment for these measures.<br>- Significant differences in improvement between groups for crawling function (t = 4.81; P < 0.001).<br>- Cognitive and language function scores, IQ, DQ, and attention factor scores increased significantly in the experimental group and were higher than in the control group after treatment (P < 0.05).	Not mentioned (the paper does not provide specific information on adverse events, adherence, or retention rates)	Not mentioned (the paper does not include any mechanistic measures such as EMG/EEG/TMS/fMRI; interlimb coupling/IHI/CPG)	Not mentioned (the paper does not provide information on fidelity checks or deviations)	- Clinical efficacy: The total treatment efficiency of the experimental group was significantly higher than that of the control group (94.44 vs. 71.87%, P = 0.012).<br>- Effectiveness: Crawling training is significantly effective in the treatment of children with cerebral palsy and notably improves crawling and cognitive functions.<br>- Clinical promotion: It is worthy of clinical promotion.	Not mentioned (the paper does not provide information on cost/resource use such as therapist time or equipment)	Not mentioned (no information on funding, COI, or trial registration is included in the paper)

Feasibility and preliminary effectiveness of a novel mobility training intervention in infants and toddlers with cerebral palsy<br>Laura A Prosser +4<br>Developmental Neurorehabilitation<br>nihms442671.pdf<br>2013 ·<br>57 citations	Single-subject research design with repeated measures during baseline, intervention, and withdrawal phases; non-controlled; non-randomized; non-blinded; pilot study	- Participant count: 5<br>- Participant age: Detailed in Table II<br>- Participant sex: Not mentioned	outpatient	Not mentioned (the paper focuses on cerebral palsy and does not provide information on stroke profile)	- Participant 1: GMFCS level III<br>- Participant 2: GMFCS level III<br>- Participant 3: GMFCS level II<br>- Participant 4: GMFCS level III<br>- Participant 5: GMFCS level I	Intervention label: Dynamic weight assistance technology; Quadruped variant: Dynamic; Main tasks: Walking, squatting, climbing stairs, crawling, getting down to and up from the floor, falling	- Session minutes: 30<br>- Sessions/week: 3<br>- Program weeks: 6<br>- Total minutes: 540	Not mentioned (the study did not include a comparator group or co-interventions)	Not mentioned (the paper does not specify an intensity metric like RPE or %HRR)	- Baseline: GMFM-66 scores and motor development rates for each participant<br>- Post-treatment: Gains in gross motor function exceeded expected rates; GMFM-66 net change scores ranged from 2.4 to 9.7<br>- Follow-up: Participants maintained treatment gains but returned to slower development rates; GMFCS classification changes observed	- Direction: Positive gains in gross motor function in four out of five participants.<br>- Magnitude: Rates of motor development during treatment were 3.8 to 15.1 times greater than during baseline.<br>- GMFM-66 net treatment change scores: Ranged from 2.4 to 9.7, exceeding minimal clinically important difference values.<br>- Participant 3 re-classified from GMFCS level II to level I.<br>- High correlation between training time and motor change (r^2 range 0.74-1.00).<br>- Strong relationship between falls and total GMFM-66 change ($r = 0.90$).	- Adverse events: Not mentioned<br>- Adherence: Average attendance and engagement rates exceeded 90%<br>- Retention: All participants maintained their treatment gains during withdrawal	Not mentioned (the paper does not include mechanistic measures such as EMG/EEG/TMS/fMRI or interlimb coupling/IHI/CPG)	- The study used a single-subject research design with repeated measures, indicating a structured approach to intervention delivery.<br>- The intervention was designed to incorporate key principles of infant motor learning and neurorehabilitation, as described in Table I.<br>- High levels of engagement and error experience during training suggest adherence to the intended protocol.<br>- Video coding was used to assess participation and error rates, indicating a method for monitoring fidelity.	- Average attendance and engagement rates exceeded 90%.<br>- The training was well-tolerated and encouraged a high level of engagement (practice) in varied motor activities with a high degree of error.<br>- Participation rate averaged 91% (range 87-95%).<br>- The findings suggest that there may be potential to alter the trajectory of motor development at a young age in CP and warrant further investigation to determine efficacy.	- Therapist time: 90 minutes per week for 6 weeks<br>- Equipment: Dynamic weight support technology (ZeroG ™)	Not mentioned (the paper does not provide information on funding, conflicts of interest, or trial registration)
The Effects of Core Stabilization Exercise on Dynamic Balance and Gait Function in Stroke Patients<br>Eun-Jung Chung +2<br>Journal of Physical Therapy Science<br>jpts-25-803.pdf<br>2013 ·<br>115 citations	Randomized, controlled, parallel design	- Core stabilization exercise group: 5 males, 3 females; Age: 44.37 (9.90)<br>- Control group: 7 males, 1 female; Age: 48.38 (9.72)	outpatient	- Type of stroke: Infarction (72.5% in core stabilization exercise group, 50% in control group), Hemorrhage (25.5% in core stabilization exercise group, 50% in control group)<br>- Chronicity/time-since: 12.88 months (core stabilization exercise group), 9.63 months (control group)<br>- Lesion side/location: Right (50% in core stabilization exercise group, 37.5% in control group), Left (50% in core stabilization exercise group, 62.5% in control group)	Not mentioned (the paper does not provide information on baseline severity using scales like NIHSS, FMA, or BBS)	- Intervention label: Core stabilization exercise<br>- Quadruped variant: Bird dog exercise (dynamic)<br>- Main tasks: Improve core stabilization for dynamic balance and gait function	30 minutes/session, 3 sessions/week, 4 weeks, 360 minutes	- Comparator: General training program (five sessions, 60 minutes per week)<br>- Co-intervention: Core stabilization exercise program (three sessions, 30 minutes per week) for the core stabilization exercise group	Not mentioned (no information on intensity metrics such as RPE or %HRR is included in the paper)	- Dynamic Balance (TUG): Baseline - Core stabilization exercise group: 33.06±18.39 sec; Control group: 30.33±12.58 sec. Post-intervention - Core stabilization exercise group: 27.64±13.73 sec; Control group: 24.85±8.76 sec.<br>- Gait Velocity: Baseline - Core stabilization exercise group: 44.83±18.83 cm/s; Control group: 37.69±11.03 cm/s. Post-intervention - Core stabilization exercise group: 58.91±18.21 cm/s; Control group: 37.39±10.11 cm/s.<br>- Gait Cadence: Baseline - Core stabilization exercise group: 74.55±13.85 steps/min; Control group: 75.90±11.73 steps/min. Post-intervention - Core stabilization exercise group: 84.07±14.00 steps/min; Control group: 77.51±10.68 steps/min.	- Dynamic balance (TUG): Decreased from 33.06±18.39 sec to 27.64±13.73 sec ($p=0.029$)<br>- Gait velocity: Increased from 44.83±18.83 cm/s to 58.91±18.21 cm/s ($p=0.024$)<br>- Cadence: Increased from 74.55±13.85 steps/min to 84.07±14.00 steps/min ($p=0.041$)<br>- Significant difference between groups: Velocity ($p=0.039$)	- Adverse events: Not mentioned<br>- Adherence: Participants practiced additional core stabilization exercises for three sessions of 30 minutes per week for four weeks.<br>- Retention: Participants were randomly divided into groups and signed informed consent, indicating structured participant management.	Not mentioned (the paper does not include any mechanistic measures such as EMG, EEG, TMS, fMRI, interlimb coupling, IHI, or CPG)	The core stabilization exercise program was implemented with fidelity, as indicated by the structured intervention and significant improvements in dynamic balance and gait parameters. No deviations or issues with fidelity were mentioned in the paper.	The paper suggests the feasibility and suitability of core stabilization exercises for stroke patients, indicating a level of acceptability. However, there are no direct quotes or data on participant satisfaction or qualitative feedback.	- Therapist time: Core stabilization exercise program for 30 minutes, three times a week; general training program for 60 minutes, five times a week.<br>- Equipment: Swiss ball, GAITRite system, SPSS statistical package.	Not mentioned (no information on funding, COI, or trial registration is included in the paper)

Effect of core stabilization exercises in addition to conventional therapy in improving trunk mobility, function, ambulation and quality of life in stroke patients: a randomized controlled trial<br>Wajeeha Mahmood +5<br>BMC Sports Science, Medicine and Rehabilitation<br>s13102-022-00452-y.pdf<br>2022 ·<br>19 citations	- Randomized controlled trial<br>- Assessor-blinded<br>- Parallel design (1:1)<br>- Concealed allocation	- Age: 45-65 years<br>- Sex: Males more in control group, females more in experimental group<br>- Count: Total 41 patients (Control group: 21, Experimental group: 20)	outpatient	- Type: Chronic ischemic stroke<br>- Chronicity/Time-since: More than 6 months, not more than 1 year<br>- Lesion side/location: Right side more affected in males, left side more affected in females	Not mentioned (the paper does not specify the use of NIHSS/FMA/BBS or similar scales for baseline severity assessment)	- Intervention label: Core stabilization training<br>- Quadruped variant: Static (multifidus activation in quadruped position)<br>- Main tasks: Abdominal drawing-in maneuver (ADIM), pelvic control exercises (anterior-posterior tilt, lateral shift, transverse rotation), bridging exercises, curl-ups, side bridging	- Control group: 40 minutes/session, 5 sessions/week, 8 weeks, total minutes = 1600<br>- Experimental group: 55 minutes/session, 5 sessions/week, 8 weeks, total minutes = 2200	Comparator: Conventional therapy; Co-interventions: Conventional physical therapy (40 min/day, 5 times/week for 8 weeks) in both groups; Experimental group received additional core stabilization training (15 min/day)	Not mentioned (the paper does not provide specific intensity metrics like RPE or %HRR)	- Trunk impairment score: Baseline (Table 2), Post-treatment (Table 3)<br>- Functional ambulation category: Baseline (Table 2), Post-treatment (Table 3)<br>- Quality of life: Baseline (Table 2), Post-treatment (Table 3)<br>- Trunk mobility (flexion, extension, side flexion, rotation): Baseline (Table 2), Post-treatment (Table 3)	- Quality of life: Core stabilization training significantly improved quality of life (161.90 ± 32.07) compared to conventional therapy (124.95 ± 37.15).<br>- Trunk flexion and extension: Statistically significant improvements in the core stabilization group.<br>- Functional ambulation: Significant improvement in the core stabilization group.<br>- Sagittal plane trunk mobility: Core stabilization training was more effective.<br>- Trunk side flexion and rotation: No significant differences between groups.	- Adverse events: Not mentioned<br>- Adherence: No crossovers between groups<br>- Retention: Not explicitly mentioned	Not mentioned (the paper does not explicitly discuss EMG, EEG, TMS, fMRI, interlimb coupling, IHI, or CPG)	- Assessor-blinded randomized control trial following CONSORT Statement Guidelines<br>- Concealment of allocation from participants and researchers<br>- Random assignment using a random number table<br>- Blinding of outcome assessors to treatment groups<br>- Appropriate statistical analysis methods<br>- No crossovers between groups<br>- Baseline comparability between groups<br>- Significant improvements in outcomes with core stabilization training<br>- Acknowledged limitations regarding long-term effects	- Quality of life was significantly improved with core stabilization training compared to conventional therapy.<br>- Core stabilization training had superior effects on quality of life, functional ambulation, and trunk impairments.<br>- The SS-QOL scale was used to measure quality of life, indicating a focus on patient satisfaction and acceptability.	- Control group: 40 minutes/day, 5 times/week for 8 weeks<br>- Experimental group: 55 minutes/day (40 minutes conventional therapy + 15 minutes core stability training), 5 times/week for 8 weeks	- Trial registration: IRCT20210614051578N 1<br>- COI: No competing interests declared<br>- Funding: Not mentioned
Use of quadrupedal step training to re-engage spinal interneuronal networks and improve locomotor function after spinal cord injury<br>Prithvi K Shah +8<br>Brain : a journal of neurology<br>awt265.pdf<br>2013 ·<br>84 citations	- Randomized<br>- Controlled<br>- Experimental study<br>- Parallel design	- Age: Adult rats<br>- Sex: Not mentioned<br>- Count: 21 rats (after excluding 2 with incomplete hemisection)	Laboratory setting	- Type: Thoracic spinal cord hemisection<br>- Chronicity/Time-since: Up to 21 days post-hemisection<br>- Lesion side/location: Right hemicord at T10 spinal segment	Not mentioned (the paper does not provide a baseline severity score using a standard scale like NIHSS/FMA/BBS)	Intervention label: Quadrupedal step-training; Quadruped variant: Dynamic; Main tasks: Locomotion on a treadmill	- Session min: 35<br>- Sessions/week: 7<br>- Program weeks: 2.5<br>- Total minutes: 595	- Comparator: Bipedal step-training (training only the hindlimbs)<br>- Co-interventions: Use of an upper body harness for bipedal step-training	Intensity metric: Treadmill speed (21 cm/s), duration (35 min/session), frequency (7 days/week), and duration of training (2.5 weeks)	- Body Function: Improved hindlimb function and coordination<br>- Activity: Enhanced locomotor recovery<br>- Timepoints: Baseline (pre-hemisection), 4 days post-hemisection, 8 days post-hemisection, 21 days post-hemisection	- Improved hindlimb function with quadrupedal step-training compared to bipedal step-training and non-trained rats.<br>- Increased excitability of spinal circuitries with quadrupedal step-training.<br>- Greater number of propriospinal labelled neurons above and below the thoracic lesion site in quadrupedally trained rats.<br>- Shorter step cycle duration and lower per cent drag duration in quadrupedal step-trained rats.<br>- Improved interlimb coordination with an 80% decrease in time lags between forelimb and hindlimb steps.<br>- Greater excitability of motor neurons with lower excitation thresholds in quadrupedal step-trained rats.	Not mentioned (the paper does not provide specific information on adverse events, adherence, or retention rates)	- Mechanism: Reorganization and re-engagement of rostrocaudal spinal interneuronal networks.<br>- Afferent feedback from forelimbs influences hindlimb activity through long descending propriospinal pathways.<br>- Interlimb coupling facilitated by chains of short propriospinal axons and ascending supraspinal pathways.<br>- Increased excitation of thoracic interneurons and bilateral symmetry in lumbosacral motor neurons.<br>- Propriospinal system integrates signals for motor neuronal pool activation.	The paper does not provide a specific numerical value for "fidelity" but describes qualitative improvements in locomotor function, indicating higher fidelity in quadrupedal step-trained rats compared to bipedal step-trained and non-trained rats.	The study provides strong evidence that actively engaging the forelimbs improves hindlimb function and coordination, indicating a high level of satisfaction with the intervention. Quadrupedal step-training is more effective than other methods, leading to improved recovery outcomes and functional coordination. The study suggests that the afferent input from the forelimbs positively influences neural substrates for coordination, contributing to overall satisfaction with the intervention's effectiveness.	Not mentioned (the paper does not provide specific details on the cost or resource use in terms of therapist time or equipment costs)	Not mentioned (no information on funding, COI, or trial registration is included in the paper)

Do core stability exercises improve upper limb function in chronic stroke patients?<br>H El-Nashar +3<br>The Egyptian Journal of Neurology Psychiatry and Neurosurgery<br>s41983-019-0087-6.pdf<br>2019 ·<br>15 citations	Randomized controlled trial, single-site, parallel design	- Age: Between 45 and 60 years<br>- Sex: Not mentioned<br>- Count: 30 participants (15 in Group A, 15 in Group B)	outpatient	- Type: Ischemic or hemorrhagic<br>- Chronicity/time-since: More than 6 months<br>- Lesion side/location: Not specified	Fugl-Meyer scale for upper limb motor performance; Modified Ashworth Scale (MAS) for spasticity	- Intervention label: Core stability exercises<br>- Quadruped variant: Dynamic<br>- Main tasks: Maintaining balance, performing cat and camel motion (spine flexion and extension)	- Session minutes: 30<br>- Sessions/week: 3<br>- Program weeks: 6<br>- Total minutes: 540	- Comparator: Conventional physical therapy program<br>- Co-interventions: Additional core muscle training for Group B	Not mentioned (the paper does not provide specific intensity metrics like RPE or %HRR)	- Upper limb function: Wolf Motor Function Test (WMFT) - baseline and post-treatment<br>- Function ability scale<br>- Time<br>- Grip strength<br>- Trunk balance: Trunk Impairment Scale (TIS) - baseline and post-treatment<br>- Static sitting balance<br>- Dynamic sitting balance<br>- Coordination	- No significant difference in upper limb function improvement between Group A and Group B.<br>- Significant improvement in trunk balance, specifically dynamic sitting balance, in favor of Group B.<br>- Core muscle training does not add significant benefits to conventional physical therapy for upper limb function but improves trunk balance.	- Adverse events: Not explicitly mentioned, but exclusion criteria suggest efforts to minimize potential risks.<br>- Adherence: All patients completed the 18 sessions over 6 weeks.<br>- Retention: All patients completed the study.	Not mentioned (the paper does not include any mechanistic measures such as EMG, EEG, TMS, or fMRI, nor does it discuss interlimb coupling, IHI, or CPG)	Not mentioned (the paper does not explicitly discuss fidelity checks or deviations from the intervention protocol)	Not mentioned (the paper does not provide specific information on participant satisfaction or acceptability beyond ethical approval and informed consent)	Not mentioned (the paper does not provide specific information on cost or resource use in terms of therapist time or equipment)	- Funding: Not mentioned<br>- COI: No competing interests<br>- Trial registration: Not mentioned
Effects of core muscles strengthening exercises with routine physical therapy on trunk balance in stroke patients: A randomized controlled trial<br>Iqra Nadeem +3<br>JPMA. The Journal of the Pakistan Medical Association<br>2023-9660.pdf<br>2024 ·<br>2 citations	- Randomized controlled trial<br>- Single-blind<br>- Controlled<br>- Non-placebo-controlled<br>- Single-site<br>- Parallel design	- Group A: 37 participants, 30 males, 7 females, mean age 56.73 years<br>- Group B: 37 participants, 27 males, 10 females, mean age 55.65 years<br>- Total participants: 74	outpatient	- Type: Ischaemic or chronic stroke<br>- Chronicity/Time-since: At least 6 months previously<br>- Lesion side/location: Group A - Right: 27%, Left: 73%; Group B - Right: 21.6%, Left: 78.4%	Not mentioned (the paper does not provide information on baseline severity using scales like NIHSS, FMA, or BBS)	- Intervention label: Core Strengthening Exercises (CSE)<br>- Quadruped variant: Not explicitly mentioned<br>- Main tasks:<br>- Step I: Trunk bracing, segmental rotation, bridging<br>- Step II: Dead bug, hamstring curls, crossed extension<br>- Step III: Side Bridge, bird dog, belly blaster	- Session minutes: RPT = 30 minutes, CSE = 15-20 minutes<br>- Sessions/week: 4<br>- Program weeks: 8<br>- Total minutes: RPT = 30 minutes/session * 4 sessions/week * 8 weeks = 960 minutes, CSE = (15-20 minutes/session) * 4 sessions/week * 8 weeks = 480-640 minutes	- Comparator: Routine physical therapy (RPT)<br>- Co-intervention for Group B: Core strengthening exercises (CSE) added to RPT	Not mentioned (the paper does not provide any intensity metrics such as RPE or %HRR)	- Trunk balance (TIS): b760 - Balance<br>- Baseline: Not significantly different between groups<br>- Week 4: Improved in both groups<br>- Post-intervention: Improved significantly in both groups, with group B better than group A<br>- Functional mobility (TUG test): d450 - Walking<br>- Baseline: Not significantly different between groups<br>- Week 4: Not explicitly mentioned<br>- Post-intervention: Improved significantly in both groups, with group B better than group A	- Trunk balance and functional mobility improved significantly in both groups post-intervention (p<0.05).<br>- Group B (CSEs + RPT) showed significantly better results than Group A (RPT alone) for both trunk balance and functional mobility (p<0.05).<br>- Baseline trunk balance scores were not significantly different between groups (p=0.92).<br>- Functional mobility scores improved significantly in both groups, but Group B had better outcomes (p<0.001).	Not mentioned (the paper does not provide specific information on safety, feasibility, adverse events, adherence, or retention)	Not mentioned (the paper does not include any mechanistic measures such as EMG, EEG, TMS, fMRI, interlimb coupling, IHI, or CPG)	- Fidelity checks: Professional physical therapist conducted sessions; standardized tools used for data collection; data analysis using SPSS 23.<br>- Deviations: Small sample size; single-centre data; difficulties with patient transportation and compliance due to low morale.	There were difficulties in terms of transportation and a lack of interest due to low morale, indicating potential issues with acceptability.	- Therapist time: RPT sessions lasted 30 minutes; CSE sessions lasted 15-20 minutes.<br>- Equipment: Not mentioned.<br>- Frequency: 4 sessions per week for 8 weeks.	Not mentioned (no information on funding, COI, or trial registration is included in the paper)
Effects of Quadruped Movement in an Individual with Chronic Effects of Quadruped Movement in an Individual with Chronic Stroke: A Case Study Stroke: A Case Study<br>Maureen Romanow +3<br>Effects of Quadruped Movement in an Individual with Chronic Strok.pdf<br>Citations unknown	Case study, observational study	- Age: 57<br>- Sex: Male<br>- Count: 1	outpatient	- Type: Left middle cerebral artery stroke<br>- Chronicity/Time-since: Occurred in 2011<br>- Lesion side/location: Left middle cerebral artery	Not mentioned (no information on primary scale scores such as NIHSS, FMA, or BBS is included in the paper)	Modified quadruped-based program (variant not specified) & constraint-induced therapy tasks	Not mentioned (the paper does not provide specific details on the dose of the intervention program)	Not mentioned (no information on comparator or co-interventions is included in the paper)	Not mentioned (no information on intensity metrics such as RPE or %HRR is included in the paper)	Not mentioned (the paper does not provide specific outcomes mapped to ICF or timepoints)	The individual with chronic stroke showed improvement following a quadruped-based intervention program. Baseline characteristics included impaired balance, use of assistive devices, and right-sided weakness. The intervention was a form of constraint-induced therapy. No specific statistical data or magnitude of improvement were reported.	Not mentioned (the paper does not provide specific information on adverse events, adherence, or retention rates)	Not mentioned (the paper does not specify any mechanistic measures such as EMG, EEG, TMS, fMRI, interlimb coupling, IHI, or CPG)	Not mentioned (the paper does not explicitly discuss fidelity checks or deviations)	Not mentioned (no explicit information on patient satisfaction or qualitative feedback is included in the paper)	Not mentioned (no information on cost/resource use is included in the paper)	Not mentioned (no information on funding, COI, or trial registration is included in the paper)

Muscle synergy analysis of eight inter-limb coordination modes during human hands-knees crawling movement<br>Chengxiang Li +6<br>Frontiers in Neuroscience<br>fnins-17-1135646.pdf<br>2023 ·<br>4 citations	- Experimental study<br>- Involves ten healthy adult participants<br>- Participants provided written informed consent<br>- Approved by an ethics review committee<br>- Structured experimental design with specific crawling tasks<br>- No randomization or blinding mentioned<br>- No control group or placebo mentioned<br>- Not observational or retrospective	- Participant count: 10<br>- Sex: 7 males, 3 females<br>- Age: 23.90 ± 0.88 years	outpatient (laboratory setting)	Not mentioned (the study involves healthy participants without any history of neuromuscular diseases or motor dysfunctions)	Not mentioned (the study involves healthy adults without neurological deficits, so baseline severity scales are not applicable)	- Intervention label: Muscle synergy extraction and analysis<br>- Quadruped variant: Kneeling<br>- Main tasks: Locomotion (crawling with specific inter-limb coordination modes)	Not mentioned (the paper does not provide specific details on the dose in terms of session minutes, sessions per week, program weeks, or total minutes)	Not mentioned (no comparator or co-intervention is mentioned in the paper)	Not mentioned (no information on intensity metrics like RPE or %HRR is included in the paper)	Not applicable (the paper does not involve interventions or assessments that would be mapped to ICF or timepoints)	- CNS controls crawling by recruiting 4 to 7 muscle synergies per participant per mode.<br>- Participants share more temporal features in synergy recruitment when crawling in the same mode.<br>- CNS uses four inter-mode shared synergies for single-limb swing modes and specific intra-mode shared synergies for two-limb swing modes.<br>- Single-limb swing modes are easier to control than two-limb modes.<br>- Simultaneous synergy recruitment may improve stride frequency.	- Adverse events: Not mentioned<br>- Adherence: Participants completed at least 8 effective crawling cycles per mode<br>- Retention: All participants completed the study as planned	- Mechanistic measure: Surface electromyography (sEMG)<br>- Rationale: Muscle synergy extraction using non-negative matrix factorization (NNMF) and hierarchical clustering for interlimb coordination analysis	Not mentioned (the paper does not explicitly provide a "Fidelity (checks/deviations)" value or section)	The study provides new evidence for the muscle synergy hypothesis in crawling, contributes to a deeper understanding of neural control mechanisms, and explains the choice of inter-limb coordination modes, indicating high satisfaction with the research outcomes.	Not mentioned (the paper does not provide specific details on the cost or resource use in terms of therapist time or equipment costs)	- Ethics approval: The Ethics Review Committee of Anhui Medical University (No. PJ 2014-08-04)<br>- Informed consent: Participants provided written informed consent<br>- Conflict of interest: No commercial or financial relationships<br>- Funding: Not mentioned<br>- Trial registration: Not mentioned
Effectiveness of kneeling training in improving mobility and balance post-stroke<br>Baojuan Cui +5<br>BMC Sports Science, Medicine and Rehabilitation<br>s13102-024-00953-y.pdf<br>2024 ·<br>0 citations	- Randomized<br>- Controlled<br>- Modified double-blind<br>- Registered on ClinicalTrials.gov	- Total participants: 67<br>- Age range: 30 to 70 years<br>- Average age: KNT group - 52 years, CVR group - 51.68 years<br>- Gender distribution: KNT group - 6 females, 25 males; CVR group - 7 females, 24 males	inpatient	- Type: Hemiplegic stroke<br>- Chronicity/Time-since: Less than 3 months since onset<br>- Lesion side/location: Right (61.29% in KNT, 48.39% in CVR), Left (38.71% in KNT, 51.61% in CVR)	- FMA-LE: KNT Group = 16.06±6.84, CVR Group = 16.58±5.75<br>- BBS: KNT Group = 26.03±12.47, CVR Group = 20.87±9.20	Kneeling Training + Kneeling & Main tasks: extending trunk, positioning arms, maintaining forward gaze, executing specific kneeling movements	- Session duration: 30 minutes<br>- Sessions per week: 6<br>- Program weeks: 4<br>- Total minutes: 720	- Comparator: Conventional treadmill walking training<br>- Co-interventions: Standard inpatient rehabilitation including physical therapy, occupational therapy, activities of daily living, and speech therapy	Not mentioned (the paper does not specify an intensity metric like RPE or %HRR)	- FMA-LE: Baseline (T0), 2 weeks (T1), 4 weeks (T2)<br>- BBS: Baseline (T0), 2 weeks (T1), 4 weeks (T2)<br>- Gait analysis (6MWT): Baseline (T0), 2 weeks (T1), 4 weeks (T2)	- Safety: The 4-week kneeling training program was established as safe.<br>- Balance: The KNT group showed more pronounced improvements in BBS scores at weeks 2 and 4 compared to the CVR group ($F = 4.263$, $P = 0.048$).<br>- Mobility: No significant disparities in FMA-LE scores between the two groups.<br>- Gait Analysis: No significant differences in most gait parameters, except for paretic step length at T2 ($P = 0.05$).	- Safety: The study established the safety of the 4-week kneeling training program with no adverse effects such as knee injuries, falls, heightened muscle tone, or evident muscle fatigue.<br>- Adherence: Participants demonstrated commendable adherence to the study, with only two individuals declining treatment due to lower back pain.<br>- Retention: 62 out of 67 eligible participants completed the treatment, indicating a high retention rate.	- Strengthening of proximal muscles associated with gait<br>- Improvement in hip control and trunk stability<br>- Exercise-induced neuroplasticity<br>- Enhanced vestibular, visual, and proprioceptive integration	High fidelity with minimal deviations; participants showed commendable adherence to the protocol, and the study was conducted in accordance with ethical standards and guidelines.	The participants demonstrated commendable adherence to the protocol, with only two patients declining treatment due to lower back pain. This suggests a high level of satisfaction or acceptability among participants. The study also posits that kneeling training has the potential to significantly augment rehabilitation therapy, indicating its acceptability for broad-scale use.	- Equipment: Kneeling training requires a large floor mat and knee pads, which are less expensive than the treadmill and safety harness used in conventional rehabilitation.<br>- Therapist time: The paper does not provide specific details on therapist time, but kneeling training may require less therapist time due to its simplicity and adaptability for home and community settings.	- Funding: Not mentioned<br>- COI: The authors declare no competing interests<br>- Trial registration: Registered on ClinicalTrials.gov under the identifier ChiCTR1900028385

Central pattern generator for locomotion: anatomical, physiological, and pathophysiological considerations<br>Pierre A Guertin<br>Front. Neur.<br>fneur-03-00183.pdf<br>2013 ·<br>224 citations	Systematic review	Not mentioned (the paper is a review and does not provide specific participant demographics)	Not applicable (the paper is a review and does not specify a clinical setting)	Not mentioned (the paper does not discuss stroke or provide a stroke profile)	Not mentioned (the paper does not provide baseline severity scores or scales such as NIHSS/FMA/BBS for any conditions)	- Intervention label: Uner Tan Syndrome (UTS)<br>- Quadruped variant: Quadrupedal gait<br>- Main tasks: Restoring bipedal walking, improving CPG coupling and activity	Not mentioned (the paper does not provide specific dosing information)	Not mentioned (the paper does not specify any comparator or co-intervention)	Not mentioned (the paper does not discuss intensity metrics such as RPE or %HRR)	Not mentioned (the paper does not provide specific outcomes mapped to ICF or timepoints)	- Plasticity changes in CPG elements contribute to pathophysiological conditions.<br>- CPG for locomotion is localized in the lumbar area of the spinal cord.<br>- Specific receptors (NMDA, 5-HT1, 5-HT7, 5-HT2A, and D1) are involved in CPG activation.<br>- Simultaneous activation of these receptors induces full locomotor effects.<br>- Epidural electrical stimulation can trigger stepping-like movements in SCI patients.<br>- The CPG shows adaptability and plasticity following SCI.<br>- Other CPGs are involved in scratching, micturition, and ejaculation.<br>- The CPG for locomotion is flexible and adaptable, offering hope for therapeutic developments.	Not mentioned (the paper does not provide specific information on safety, adverse events, adherence, or retention rates)	- Mechanistic measures: Pharmacological manipulations using receptor ligands (agonists and antagonists) to understand CPG organization.<br>- Rationale: Identification of specific receptors (NMDA, 5-HT1, 5-HT7, 5-HT2A, D1) involved in CPG activation using selective antagonists and genetically manipulated animals.<br>- Interlimb coupling/IHI/CPG: Dual immunohistochemical experiments showing locomotor activity-related receptors on CPG neurons in the lumbar cord.	The paper discusses the adaptability and flexibility of the CPG for locomotion, indicating that it can undergo changes in response to SCI. While it does not provide a specific measure or value for "fidelity," it suggests that the CPG can maintain some level of functionality despite deviations from its normal state.	Not mentioned (the paper does not provide information on acceptability or satisfaction)	- Electrical stimulation of extrapyramidal tracts: Requires specialized equipment and therapist time.<br>- Direct stimulation of the CPG: Requires specialized equipment and possibly therapist time.<br>- Pharmacological aids (e.g., adrenergic α1 agonists): Involves medication costs.<br>- Rehabilitation approaches (e.g., BWSTT): Requires therapist time and possibly specialized equipment.<br>- Development of Spinalon™: Potential future resource use for medication development and administration.	The author declares financial competing interests in Nordic Life Science Pipeline.
Use it or lose it? Effects of age, experience, and disuse on crawling<br>Whitney G Cole +4<br>Developmental Psychobiology<br>nihms-1006698.pdf<br>2020 ·<br>29 citations	- Non-controlled study<br>- Comparative study design<br>- Mixed cross-sectional and longitudinal study design<br>- Retrospective component<br>- Observational study	- Infants: 34 (15 boys, 11 girls, 5 boys, 3 girls)<br>- Children: 27 (14 boys, 13 girls)<br>- Young Adults: 13 (5 men, 8 women)<br>- Adults with Uner Tan Syndrome: 5 (1 man, 4 women)	outpatient	- Type: Uner Tan Syndrome<br>- Chronicity/Time-since: Lifelong since infancy<br>- Lesion side/location: Cerebellar abnormalities	Not mentioned (the paper does not include any information on baseline severity scales like NIHSS/FMA/BBS)	- Intervention label: Habitual practice vs. disuse of crawling<br>- Quadruped variant: Moving on all fours (static/dynamic/locomotion/kneeling)<br>- Main tasks: Crawling on hands and knees, crawling on hands and feet	Not mentioned (the paper does not provide specific information on session duration, sessions per week, program weeks, or total minutes)	- Comparator: Four groups of participants with different amounts of habitual practice (infants, children, young adults, and adults with Uner Tan Syndrome).<br>- Co-interventions: None mentioned beyond the natural differences in habitual practice and disuse.	Not mentioned (the paper does not provide traditional intensity metrics like RPE or %HRR)	- Body function: Gait types, speed, limb support<br>- Activity: Crawling ability<br>- Timepoints: Baseline (initial assessment), Post (assessment after disuse or continued practice)	- Habitual crawlers showed similarities in gait patterns and speed despite differences in practice and body size.<br>- Habitual crawlers predominantly used trot-like gaits; rusty crawlers showed a variety of gait patterns.<br>- Children crawled faster and used fewer limbs on the ground compared to other groups.<br>- Old crawling patterns were retained despite disuse; new ones were added.<br>- Experienced infants crawled faster than less experienced ones ($\chi^2 = 6.24$, $p = 0.01$ for speed; $\chi^2 = 20.36$, $p < 0.001$ for cadence).<br>- Young adults crawled faster than infants; children were the fastest (M = 0.58 body lengths/second for children; M = 0.29 for infants; M = 0.40 for young adults).<br>- Statistical analyses: $F(2,70) = 81.03$, $p < 0.001$ for speed; $F(2,58) = 24.96$, $p < 0.001$ for normalized speed; $F(2,70) = 15.51$, $p < 0.001$ for cadence.<br>- Infants had higher limb support values than children and adults.	Not mentioned (the paper does not provide specific information on adverse events, adherence, or retention)	Not mentioned (the paper does not include mechanistic measures such as EMG/EEG/TMS/fMRI or interlimb coupling/IHI/CPG)	- Infants: Switched gait types 0-6 times within a sequence.<br>- Children: Switched gait types 0-4 times within a sequence.<br>- Young Adults: Switched gait types 0-2 times within a sequence.<br>- UTS Group: Maximum number of switches observed was 28.	- Satisfaction: The study found that disuse did not harm crawling ability, and some features were even gained or altered.<br>- Qualitative quotes: "Nothing was lost with disuse, but some features of crawling were gained or altered." "Disuse did not harm crawling ability." "Rusty child and young adult crawlers performed as well as-or better than-habitual crawlers." "Disuse comes with no penalty-at least on some critical metrics."	Not mentioned (the paper does not provide information on therapist time or equipment costs)	Not mentioned (the paper does not provide information on funding, COI, or trial registration)

											- Children used fewer limbs for support than young adults and the UTS group.<br>- Young adults were more consistent in gait patterns than infants and children (F(2,73) = 3.69, p = 0.04 for switches/stride).						
Interlimb neural coupling: Implications for poststroke hemiparesis Le couplage neuronal inter-membres : implications pour l'he ´mipare ´sie suite a `un accident vasculaire ce ´re ´bral<br>K N Arya +1<br>1-s2.0-S18770657140 1745X-main.pdf<br>Citations unknown	Systematic review	- 16 healthy subjects: age range 18-29 years<br>- 13 healthy subjects: 5 men, 8 women; age 22.1 AE 3.2 years<br>- 21 healthy individuals: age 30.4 AE 8.6 years<br>- 8 poststroke subjects: 50% men; age 60.4 AE 11.1 years<br>- 10 healthy controls: 40% men; age 60.2 AE 8.9 years<br>- 10 chronic stroke subjects: no specific age or sex details<br>- 10 healthy subjects: no specific age or sex details<br>- 16 poststroke subjects: no specific age or sex details<br>- 3 chronic stroke patients: no specific age or sex details	Not mentioned (the paper does not specify the setting for rehabilitation)	- Type: Not mentioned<br>- Chronicity/Time-since: Chronic, more than 12 months<br>- Lesion side/location: Not mentioned	Not mentioned (the paper does not provide specific baseline severity data or scales like NIHSS/FMA/BBS)	- Intervention label: Bilateral upper and lower limb movements, Bimanual upper limb training, BWSTT, Elliptical machine training<br>- Quadruped variant: Static (bimanual training), Dynamic (BWSTT, elliptical machine)<br>- Main tasks: Reaching and grasping bilateral objects, carrying objects, reading the newspaper	- Session duration: 15 minutes (for adaptation session)<br>- Sessions/week: 3 days/week<br>- Program weeks: 4 weeks<br>- Total minutes: Not explicitly mentioned	- Comparator: Bilateral training vs. standard management<br>- Co-interventions: Bilateral upper and lower limb movements, BWSTT with split-belt and arm swing facilities, virtual training, robotic therapy	Not mentioned (the paper does not explicitly mention intensity metrics like RPE or %HRR)	- Improved motor function through bilateral upper and lower limb movements<br>- Enhanced gait symmetry and speed with split-belt treadmill training<br>- Significant impairment in dual-task gait variables<br>- Improved walking speed with supported arm movements<br>- Decreased shoulder muscle activity with simultaneous upper and lower limb cycling<br>- Increased shoulder flexor activity in mild paretic subjects with non-paretic upper extremity loading	- Incorporating bilateral upper and lower limb movements enhances motor function and neural plasticity.<br>- Repetitive bimanual movements improve motor function.<br>- Stronger activation of bilateral proximal upper extremity muscles in the paretic side during tibial nerve stimulation.<br>- Significant increase in flexor activities of paretic fingers and elbow during walking (P < 0.001 and P < 0.005).<br>- Strong interlimb coupling impairs rhythmical bilateral coordination of the affected limb.<br>- Significant impairment in dual-task gait variables among stroke subjects.<br>- Split-belt treadmill training enhances gait symmetry and speed (lasting effects 1-month post-training).<br>- Significant deficits in antiphase tasks among stroke subjects.	- Safety: The use of the elliptical machine and dual-task exercises were safe.<br>- Feasibility: The interventions were feasible for patients to participate in.<br>- Adverse events: Not mentioned.<br>- Adherence: Improved functional mobility, endurance, and balance suggest good adherence.<br>- Retention: The interventions were effective in improving gait variables except temporal asymmetry.	- EMG: Used to record muscle activity in healthy subjects during interlimb coordination tasks.<br>- EEG: Demonstrated specific activation in SMA during bimanual movements.<br>- Interlimb Coupling: Discussed in relation to muscle activation and neural coordination.<br>- IHI: Mentioned as part of the neural mechanisms influencing interlimb coordination.<br>- CPG: Central pattern generators play a role in rhythmic movements and interlimb coordination.<br>- Kinematic Variables and Muscle Activity: Assessed in poststroke subjects to understand motor deficits.	Not mentioned (the paper does not explicitly discuss "Fidelity (checks/deviations)")	The paper suggests that incorporating bilateral upper and lower limb movements and repetitive bimanual coordinated movements could enhance motor function and neural plasticity, potentially leading to increased patient satisfaction with rehabilitation outcomes. The authors emphasize the need to re-evaluate existing methods and develop new interventions based on the coupling concept, which could improve patient satisfaction by enhancing motor recovery and functional improvement.	Not mentioned (the paper does not provide specific details on cost or resource use)	Not mentioned (the paper does not provide information on funding, COI, or trial registration)
Measurement and Analysis of Human Infant Crawling for Rehabilitation: A Narrative Review<br>Qi L Xiong +4<br>Frontiers in Neurology<br>fneur-12-731374.pdf<br>2021 ·<br>10 citations	Not mentioned (the paper is a narrative review)	- Yozu et al.: 8 healthy infants<br>- Patrick et al.: 26 healthy infants<br>- Xiong et al.: 20 healthy infants, 47 infants with developmental delays<br>- Other studies: Various counts mentioned in the table	Not mentioned (the paper does not specify the setting for the measurements or interventions discussed)	Not mentioned (the paper does not provide specific details about the stroke profile for infants with CP)	Not mentioned (the paper does not specify a primary scale like NIHSS/FMA/BBS for baseline severity)	- Intervention label: Crawling training<br>- Quadruped variant: Locomotion<br>- Main tasks: Promote locomotor function, assess motor development, improve gait	Not mentioned (the paper does not provide specific details on the dose of the intervention)	Not mentioned (the paper does not explicitly mention a comparator group or co-interventions in the context of a controlled study)	- Power<br>- Root mean square<br>- Mean absolute value of sEMG signals<br>- Activation level of muscles (e.g., quadriceps, tibialis anterior, triceps, posterior deltoid)<br>- Coactivation index (CI)	Not mentioned (the paper does not explicitly provide a detailed mapping of outcomes to the ICF or specify timepoints for these outcomes)	- Early intervention has positive effects on motor development.<br>- Robotic assistants promote crawling in infancy.<br>- Strong correlation between stance duration and crawling velocity.<br>- Quantification of phase durations based on crawling velocity.<br>- Muscle coactivation patterns and interlimb joint synergy metrics are important for motor development.<br>- Indices proposed for evaluating muscle activity and kinematic features in crawling.	Not mentioned (the paper does not provide specific information on safety, adverse events, adherence, or retention related to crawling interventions)	sEMG for muscle activity monitoring, muscle synergy analysis, interlimb coordination (diagonal coupling), central pattern generators (CPGs), CNS motor command generation, corticospinal tract maturation	Not mentioned (the paper does not explicitly mention "Fidelity (checks/deviations)" in the text)	The attachment of sensors and markers may cause discomfort and hinder natural crawling behavior, potentially reducing acceptability. However, there are no explicit satisfaction or qualitative quotes provided in the paper.	- Equipment: Motion capture systems, surface electromyography (sEMG), computer vision technology, crawling training devices, skateboard devices, orthoses, crawl and gait stimulators.<br>- Therapist time: Significant time required for setup, data collection, and analysis using these technologies and devices.	Not mentioned (no information on funding, COI, or trial registration is included in the paper)

Interlimb Coordination in Human Crawling Reveals Similarities in Development and Neural Control With Quadrupeds<br>Susan K Patrick +2<br>Journal of Neurophysiology<br>patrick-et-al-2009-interlimb-coordination-in-human-crawling-reveals-similarities-in-development-and-neural-control-with.pdf<br>2009 ·<br>96 citations	Observational study with experimental manipulation of variables; non-randomized selection of subjects; laboratory setting with controlled conditions; no control group or randomization mentioned.	- Infants: 26, Age: 7-13 months<br>- Adults: 7, Age: Not specified<br>- Sex: Not mentioned	outpatient/laboratory	Not mentioned (the paper does not provide information on stroke profile, type, chronicity/time-since, or lesion side/location)	Not mentioned (the paper does not include any information on baseline severity scales such as NIHSS, FMA, or BBS)	- Intervention label: Crawling<br>- Quadruped variant: Hands-and-knees crawling<br>- Main tasks: Crawling on a treadmill with varying speeds, manipulating mechanical factors (belt speed, width of base, limb length), performing cognitive tasks to ensure automaticity of crawling	Not mentioned (the paper does not provide information on the number of sessions per week, total program weeks, or total minutes)	- Comparator: Quadrupeds<br>- Co-interventions: Manipulation of belt speed, width of base, and limb length in adults; unweighting and limb length in infants	Not mentioned (the paper does not include specific intensity metrics like RPE or %HRR)	Not mentioned (the paper does not provide outcomes mapped to ICF or specify timepoints such as baseline, post, or follow-up)	- Infants primarily used a trot-like gait, while adults used both trot-like and pace-like gaits.<br>- At lower speeds, limb movements were more equally spaced with no clear pairing; at higher speeds, no running symmetrical gaits were observed.<br>- Widening stance prevented adults from using pace-like gaits; lengthening limb length prevented trot-like gaits.<br>- Limb length and unweighting had no effect on infant coordination.<br>- Adults showed greater flexibility in coordination compared to infants.<br>- Weak relationship between crawling speed and coordination pattern, with more variability at lower speeds.	- Adverse events: Not mentioned<br>- Adherence: Informed consent and adherence to ethical guidelines<br>- Retention: Not mentioned	- EMG: Surface bipolar EMG recordings were collected but not presented.<br>- Interlimb coupling: Implied flexibility in neural coupling between limbs.<br>- IHI/CPG: Not mentioned.<br>- EEG/TMS/fMRI: Not mentioned.	- Infants: Primarily trot-like gait with less flexibility in coordination.<br>- Adults: Greater variety in coordination (trot-like and pace-like gaits) with flexibility influenced by mechanical factors.<br>- Deviations: Absence of running symmetrical gaits at higher speeds; galloping observed in one adult.<br>- Checks: Smooth transitions between coordination patterns suggest shared neural circuitry.	The study suggests that human crawling shares similarities with quadrupedal locomotion, indicating common underlying mechanisms. Adults show flexibility in coordination patterns, while infants have restricted options due to nervous system immaturity. The coordination patterns in humans are similar to those in nonprimate quadrupeds, suggesting shared circuitry.	Not mentioned (the paper does not provide information on therapist time or equipment costs)	Not mentioned (no information on funding, COI, or trial registration is included in the paper)
