# Supplementary material for: Quadrupedal training approaches in post-stroke rehabilitation: a scoping review of evidence, mechanisms, and clinical applications
Source: Front Syst Neurosci. 2026 Apr 8;20:1773330. doi: 10.3389/fnsys.2026.1773330 (PMC13099906; doi:10.3389/fnsys.2026.1773330)
Supplement: Supplementary file 3 [file Data_Sheet_3.docx]

**TABLE S3** | Evidence mapping: dosing, supervision, progression, and role of each included study.

| **Evidence tier** | **Study** | **Population / model** | **QT-related task or posture** | **QT minutes / session** | **Sessions / week** | **Weeks** | **Weekly QT minutes** | **Total QT minutes** | **Supervision** | **Progression criteria** | **How used in this review** |
| --- | --- | --- | --- | --- | --- | --- | --- | --- | --- | --- | --- |
| Stroke clinical | Chung et al., 2013 | Chronic stroke; outpatient | Dynamic QT (core / contralateral tasks) | NR | 3 | 4 | NR | NR | Therapist-supervised | Implicit progression (task difficulty); explicit rules NR | Clinical outcome synthesis |
| Stroke clinical | El-Nashar et al., 2019 | Chronic stroke; outpatient | Dynamic QT (cat–camel, bridging) | NR | 3 | 6 | NR | NR | Therapist-supervised | Repetitions / hold duration reported; criteria NR | Clinical outcome synthesis |
| Stroke clinical | Mahmood et al., 2022 | Chronic ischemic stroke; outpatient | Static + dynamic QT | 15 | 5 | 8 | 75 | 600 | Therapist-supervised | Progressive reps and hold duration | Clinical outcome synthesis |
| Stroke clinical | Nadeem et al., 2024 | Chronic stroke; outpatient | Dynamic QT (progressive core/QT) | 15–20 | 4 | 8 | 60–80 | 480–640 | Therapist-supervised | Stage-based progression (I–III) | Clinical outcome synthesis |
| Stroke clinical | Zhang et al., 2024 | Subacute stroke; inpatient | Kneeling QT | 30 | 6 | 4 | 180 | 720 | Therapist-assisted | Speed and HR-guided intensity | Clinical outcome synthesis |
| Stroke clinical | Pascal et al., 2022 (case) | Chronic stroke; outpatient | Locomotor QT (crawling) | 60 | 2 | 8 | 120 | 960 | Therapist-supervised | Progressive sequencing complexity | Clinical outcome synthesis |
| Mechanistic / translational | Shah et al., 2013 | SCI (rat model) | Quadrupedal step training | NR | NR | NR | NR | NR | Assisted / experimental | Speed and task complexity | Mechanistic plausibility |
| Mechanistic / translational | Li et al., 2023 | Healthy adults | Hands-and-knees crawling | NR | NR | Single session | NR | NR | Lab-supervised | Task condition manipulation | Mechanistic plausibility |
| Mechanistic / translational | Xiong et al., 2021 | Infants | Crawling measurement paradigms | NR | NR | NR | NR | NR | Observational | Developmental staging | Mechanistic context |
| Mechanistic / translational | Arya & Pandian, 2014 | Review | Interlimb coupling | NA | NA | NA | NA | NA | NA | Narrative synthesis | Mechanistic context |
| Mechanistic / translational | Guertin, 2013 | Review | CPG / locomotor networks | NA | NA | NA | NA | NA | NA | Narrative synthesis | Mechanistic context |
| Mechanistic / translational | Frigon, 2017 | Review | Interlimb coordination | NA | NA | NA | NA | NA | NA | Narrative synthesis | Mechanistic context |
| Mechanistic / translational | Vanicek et al., 2013 | Review | Postural control / COP | NA | NA | NA | NA | NA | NA | Narrative synthesis | Mechanistic context |

*QT, quadruped training; NR, not reported; NA, not applicable; PT, physical therapy; HR, heart rate; SCI, spinal cord injury; CPG, central pattern generator; COP, centre of pressure.QT minutes/session refers to time explicitly attributable to quadruped-derived postures or tasks. When posture-specific exposure could not be isolated from broader experimental or clinical protocols, dose values are reported as NR. Mechanistic and translational records are included for biological plausibility and implementation context only and were not used to infer clinical effectiveness.*
